# Human papillomavirus type 16 antagonizes IRF6 regulation of IL-1β

**DOI:** 10.1371/journal.ppat.1007158

**Published:** 2018-08-08

**Authors:** Michelle Ainouze, Pauline Rochefort, Peggy Parroche, Guillaume Roblot, Issam Tout, François Briat, Claudia Zannetti, Marie Marotel, Nadege Goutagny, Philip Auron, Alexandra Traverse-Glehen, Aude Lunel-Potencier, Francois Golfier, Murielle Masson, Alexis Robitaille, Massimo Tommasino, Christine Carreira, Thierry Walzer, Thomas Henry, Katia Zanier, Gilles Trave, Uzma Ayesha Hasan

**Affiliations:** 1 Centre International de recherche en Infectiologie, CIRI, Inserm, U1111, Lyon, France; 2 Université Claude Bernard Lyon 1, Lyon, France; 3 CNRS, UMR5308, Lyon, France; 4 École Normale Supérieure de Lyon, Univ Lyon, France; 5 Hospices Civils de Lyon, France; 6 Cancer Research Centre of Lyon, INSERM U1052-CNRS UMR5286, Lyon, France; 7 Duquesne University, Pittsburgh, Pennsylvania, United States of America; 8 IGBMC, UMR 7104-U964, ILLKIRCH, France; 9 IARC, Lyon, France; University of Wisconsin Madison School of Medicine and Public Health, UNITED STATES

## Abstract

Human papillomavirus type 16 (HPV16) and other oncoviruses have been shown to block innate immune responses and to persist in the host. However, to avoid viral persistence, the immune response attempts to clear the infection. IL-1β is a powerful cytokine produced when viral motifs are sensed by innate receptors that are members of the inflammasome family. Whether oncoviruses such as HPV16 can activate the inflammasome pathway remains unknown. Here, we show that infection of human keratinocytes with HPV16 induced the secretion of IL-1β. Yet, upon expression of the viral early genes, IL-1β transcription was blocked. We went on to show that expression of the viral oncoprotein E6 in human keratinocytes inhibited IRF6 transcription which we revealed regulated IL-1β promoter activity. Preventing E6 expression using siRNA, or using E6 mutants that prevented degradation of p53, showed that p53 regulated IRF6 transcription. HPV16 abrogation of p53 binding to the IRF6 promoter was shown by ChIP in tissues from patients with cervical cancer. Thus E6 inhibition of IRF6 is an escape strategy used by HPV16 to block the production IL-1β. Our findings reveal a struggle between oncoviral persistence and host immunity; which is centered on IL-1β regulation.

## Author summary

Oncoviruses block innate immune responses to persist in the host. However, to avoid viral persistence, the immune response attempts to clear the infection. IL-1β is a pro-inflammatory cytokine produced by the inflammasome pathway. Whether oncoviruses such as human papillomavirus (HPV) can activate the inflammasome remains to be explored. We demonstrated that keratinocytes, the host cell type for papillomaviruses, when infected with HPV16 induced IL-1β transcription and secretion. Yet, upon expression of the viral oncoprotein E6, IL-1β transcription was blocked. E6 expression inhibited IRF6 transcriptional regulation of the IL-1β promoter. Preventing E6 expression, or its ability to degrade p53, restored the ability of IRF6 to bind to the IL-1β promoter. HPV16 abrogation of p53, IRF6 and IL-1β expression was fully confirmed in cervical cancer cells and tissues from patients. These data highlight the equilibrium between the host innate immune rheostat and viral immune escape.

## Introduction

The innate immune system is the first line of defense in response to danger signals from microbial invasion or tissue injury. Viruses are sensed by several immune receptors that activate signaling pathways leading to cytokine production. Many oncogenic viruses can deregulate several immune-related pathways which guarantee a persistent infection. High-Risk Human Papilloma Viruses (HR HPV) are the etiological factor of cervical as well as certain head and neck cancers and is responsible for 20% of all human cancers linked to infection [1]. Persistence and progression of the disease are achieved by deregulating both cellular and immune defense mechanisms. Among the HR types, HPV16 is the most prevalent type in premalignant and malignant cervical lesions [2]. HPV16 viral oncoproteins E6 and E7 can target many cellular proteins such as binding and degrading the tumor suppressors’ p53, and pRb, respectively. In parallel E6 and E7 are able to deregulate several innate immune-related pathways that block cytokine and chemokine production, antigen presentation, and adherence molecules [3]. Recently Lau et al., showed that E7 from HPV18 suppresses the cGAS pathway by inhibiting the adapter protein STING [4]. Similarly, some antiviral genes induced by interferons such as IFIT1, MX1 and the innate sensors RIG-I, TLR3 and TLR9 are also inhibited by HPV [5,6]. Indeed, Niebler et al., and Karim et al., have shown that HPV is capable of blocking IL-1β [7,8]. On the flip side, host cells have strategies to thwart viral immune escape.

IL-1β is crucial in host-defenses towards infection and injury. Our current understanding is that regulation of IL-1β is controlled by two checkpoints: 1. The activation and translocation of the nuclear factor-κB (NF-κB) which initiates the transcription of the pro-IL-1β gene. 2. Post-translational regulation of pro-IL-1β into its cleaved form by the inflammasome cytosolic multi-protein complex. The inflammasome complex consists of an innate pathogen recognition receptors such as the nucleotide-binding domain and leucine-rich repeat pyrin domain 3 (NLRP3) or absent in melanoma 2 (AIM2). Upon viral recognition the inflammasome sensor recruits the apoptosis-associated, speck-like protein containing a carboxy-terminal CARD (ASC). Caspase-1 is activated within the inflammasome multiprotein complex through interaction with ASC that bridges NLRP3 or AIM2. The activation of caspase-1 is associated with pyroptosis, a form of programmed cell death distinct from apoptosis, as well as the cleavage of the proinflammatory cytokines IL-1β and IL-18. Once released, mature IL-1β and IL-18 signal to their target cells, thus allowing the expansion of innate and adaptive immune responses. NLRP3 inflammasomes are activated by viruses such as adenovirus [9], vaccinia virus [10], and hepatitis C virus (HCV) [11]. The AIM2 inflammasome has been shown to detect vaccinia virus [12] and murine CMV [13]. Whether the inflammasome plays a protective role against HPV16 remains to be investigated.

Here we demonstrate that HPV16 induces the secretion of IL-1β from human keratinocytes. IL-1β produced from HPV16 infected keratinocytes blocked gene viral transcription. However, inhibition was lost after 8h due to the ability of the viral oncoprotein E6 (16E6) to inhibit IL-1β transcription. A 16E6 protein binding domain essential for p53 degradation played a crucial role in regulating IL-1β transcription. 16E6 blocked the p53 transcriptional regulation of Interferon Regulatory Factor 6 (IRF6), which we found was essential for IL-1β promoter activity. The identification of this inhibitory transcriptional loop represents an undiscovered mechanism of oncoviral immune hijacking in the infected host cell.

## Results

### HPV16 induces the transcription and secretion of IL-1β

**Fig 1:**
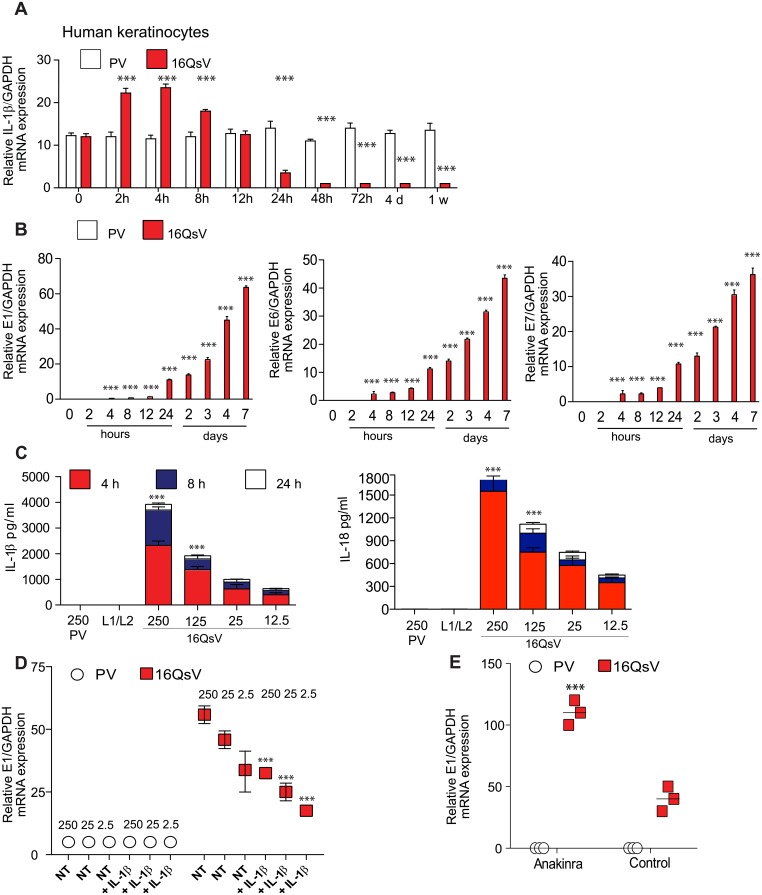
HPV16 induces transient IL-1β secretion by keratinocytes. (A) Human primary keratinocytes were treated as indicated with 16QsV or PsV (at 200 viral genome equivalents (v.g.e) per cell). IL-1β transcripts were determined by RT-qPCR. n = 5. (B) As in A, E1, E6 and E7 mRNA relative levels were determined by RT-qPCR. n = 4. (C) Human keratinocytes were treated at 4, 8 and 24 h with 16QsV at different v.g.e per cell. Supernatants were harvested and IL-1β or IL-18 production was measured by ELISA. PsV or L1/L2 fractions were added as controls. n = 5. (D) Human keratinocytes were treated with 16QsV or PsV at decreasing v.g.e per cell for 24 h ± recombinant IL-1β (200pg/ml). Cells were harvested and E7 mRNA levels were measured by RT-qPCR. n = 5. (E) Human keratinocytes were treated with 16QsV or PsV (200 v.g.e) for 24 h ± IL-1R inhibitor (Anakinra). Cells were harvested and E1 mRNA levels were measured by RTqPCR. qPCR. n = 5. Data are representative of n independent experiments performed. Shown are the mean ± SEM with ***, P < 0.0001, based on a two way ANOVA test.

We first determined whether inflammasome activation could be achieved in normal human keratinocytes, the host of HPV infection. Addition of poly dA:dT (an AIM2 activator) or Nigericin (an NLRP3 agonist) led to the secretion of IL-1β (S1 Fig). Of note, the induction of pro-IL-1β did not require the first check point signal (S1A Fig). Pro-IL-1β is constitutively expressed in human keratinocytes and has been previously described by Sand et al., and Zepter et al [14,15]. We next tested whether HPV16 induced IL-1β gene expression in human keratinocytes. To do this, we generated HPV16 Quasivirions (16QsV) that closely resemble the natural virus as well control Pseudovirions (PsV). 16QsV are viral particles that contain the full viral genome of HPV16 encaspidated by the viral late proteins L1 and L2 (L1/L2). PsV are viral particles that contain GFP DNA encaspidated by L1/L2 [6]. Infection in keratinocytes with 16QsV up to 4h led to an increase of IL-1β transcripts (Fig 1A). However, post 8h infection, IL-1β transcription decreased (Fig 1A). The level of IL-1β gene expression inversely correlated to viral gene transcription (Fig 1B). Furthermore, primary keratinocytes infected with 16QsV induced IL-1β or IL-18 secretion at 4h but not at 24h (Fig 1C). 16QsV induction of IL-1β depended on caspase-1 activity (S1B Fig). Pyroptosis was also induced by 16QsV as measured by lactate dehydrogenase activity (S1C Fig). We did not observe IL-1β secretion when PsV or when extracts of the late proteins L1/L2 was added to keratinocytes (Fig 1C). These data suggest that 16QsV can induce caspase -1 dependent IL-1β, IL-18 as well as pyroptosis during the early phases of infection.

### IL-1β production is blocked by the viral oncoproteins 16E6 and E7

**Fig 2:**
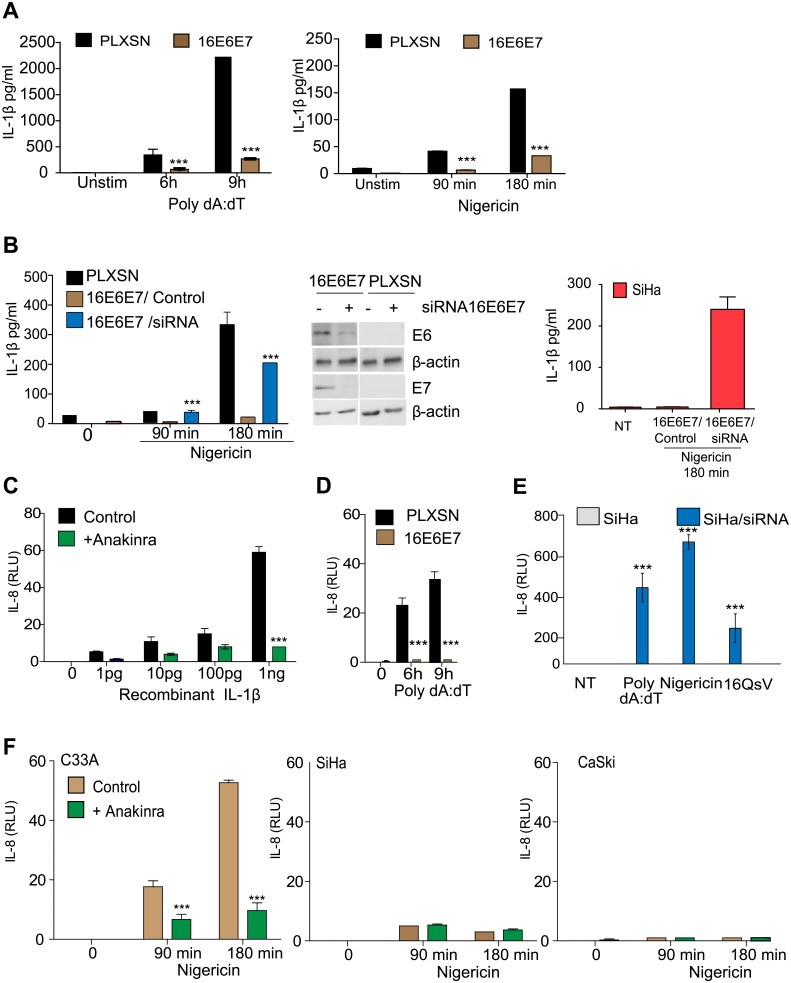
16E6E7 block IL-1β production in primary human keratinocytes and in cervical cancer derived cells lines. (A) Analysis of the IL-1β production by ELISA in human keratinocytes transduced with pLXSN or 16E6E7 stimulated with nigericin or poly dA:dT. n = 10. (B) Human keratinocytes transduced with pLXSN or 16E6E7 transfected with a siRNA targeting 16E6E7 (+) or the scramble control (-). Cells were stimulated with the NLRP3 ligand nigericin and IL-1β secretion was measured by ELISA. Middle, western blot of E6 or E7 siRNA efficacy on 16E6E7 or PLXSN transduced cells. n = 4. Left SiHa cell were treated with a siRNA targeting 16E6E7 (+) or the scramble control (-). Cells were stimulated with the NLRP3 ligand nigericin and IL-1β secretion was measured by ELISA. n = 4. (C) IL-8 bioassay: HEK293T cells transiently expressing the IL-8 promoter linked to luciferase gene were treated with increasing concentrations of recombinant IL-1β ± Anakinra. Twenty four h post treatment cells were harvested and luciferase activity was measured. n = 4. (D) IL-8 bioassay using supernatants from human keratinocytes transduced with pLXSN or 16E6E7± AIM2 ligand poly dA:dT. n = 4. (E) Cervical cancer cells (SiHa) were transfected with a siRNA targeting 16E6E7 or the scramble control. The cells were stimulated with the NLRP3 ligand nigericin, AIM2 ligand poly dA:dT or 16QsV (200 v.g.e per cell) and IL-1β was measured by ELISA. n = 4. (F) IL-8 bioassay using supernatants from cervical cancer cell lines ± nigericin. n = 6. Data are representative of n independent experiments performed in triplicate. Shown are the mean ± SEM with ***, P < 0.0001, based on a two way ANOVA test. For immunoblotting data, 1 out of n = 3 experiments is shown.

IL-1β has been shown to block HBV replication in human hepatocytes [16]. Therefore we evaluated whether IL-1β could inhibit HPV16 viral gene transcription. Primary keratinocytes were infected with 16QsV or PsV ± recombinant IL-1β. We observed that IL-1β blocked 16QsV viral expression as measured by E1 transcripts (Fig 1D). This effect was reversed when we blocked the IL-1 receptor using Anakinra (Fig 1E). The viral oncoproteins E6 and E7 inhibit several innate immune pathways such as TLR9, STING and IRF signaling [4,6,17]. Based on these reports we hypothesized that E6 and E7 were responsible for the inhibition of IL-1β. To test this, human primary keratinocytes were transduced with recombinant retrovirus expressing HPV16 E6 and E7 (16E6E7) or with the empty vector control (pLXSN). 16E6E7 blocked both AIM2 and NLRP3-mediated secretion of IL-1β (Fig 2A). Furthermore, knock down of the viral oncoproteins using siRNA targeting16E6E7 restored the ability of cells to produce IL-1β (Fig 2B). In the epidermis, keratinocytes are the first cells to be encountered by external stimuli to induce IL-1β which in turn stimulates IL-8 secretion by human dermal fibroblasts [18]. We established an IL-8 bioassay in which addition of recombinant IL-1β induced IL-8 promoter activity of the luciferase gene in HEK293 cells (Fig 2C). Specificity of the assay was controlled using IL-1R inhibitor (Anakinra) (Fig 2C). Supernatants that were derived from AIM2 stimulated primary human keratinocytes induced the expression of the IL-8 luciferase gene. However, supernatants derived from AIM2 stimulated 16E6E7 cells failed to induce IL-8 transcription (Fig 2D). Furthermore, knock down of the viral oncoproteins using siRNA for 16E6E7 restored the ability of a cervical cancer-derived cell line (SiHa HPV16+) to produce IL-1β in response to Nigericin, poly dA:dT and 16QsV (Fig 2E). Thus 16E6E7 oncoproteins block IL-1β secretion. We corroborated our findings using supernatants from cervical cancer cell lines that were stimulated with the NLRP3 ligand. We observed that supernatants from the cervical cell line C33A (HPV-) stimulated with nigericin induced IL-8 luciferase activity (Fig 2F). Furthermore, Anakinra blocked IL-8 gene induction from supernatants derived from C33A cells stimulated with the NLRP3 ligand (Fig 2F). In contrast, supernatants taken from SiHa and CaSki cells (HPV16+) that were stimulated with nigericin failed to induce IL-8 promoter activity (Fig 2F). In summary, we have demonstrated the ability of HPV16 E6 and/or E7 to block IL-1β paracrine induction of IL-8 transcription.

### HPV16E6E7 abrogates mRNA expression of pro-IL-1β

**Fig 3:**
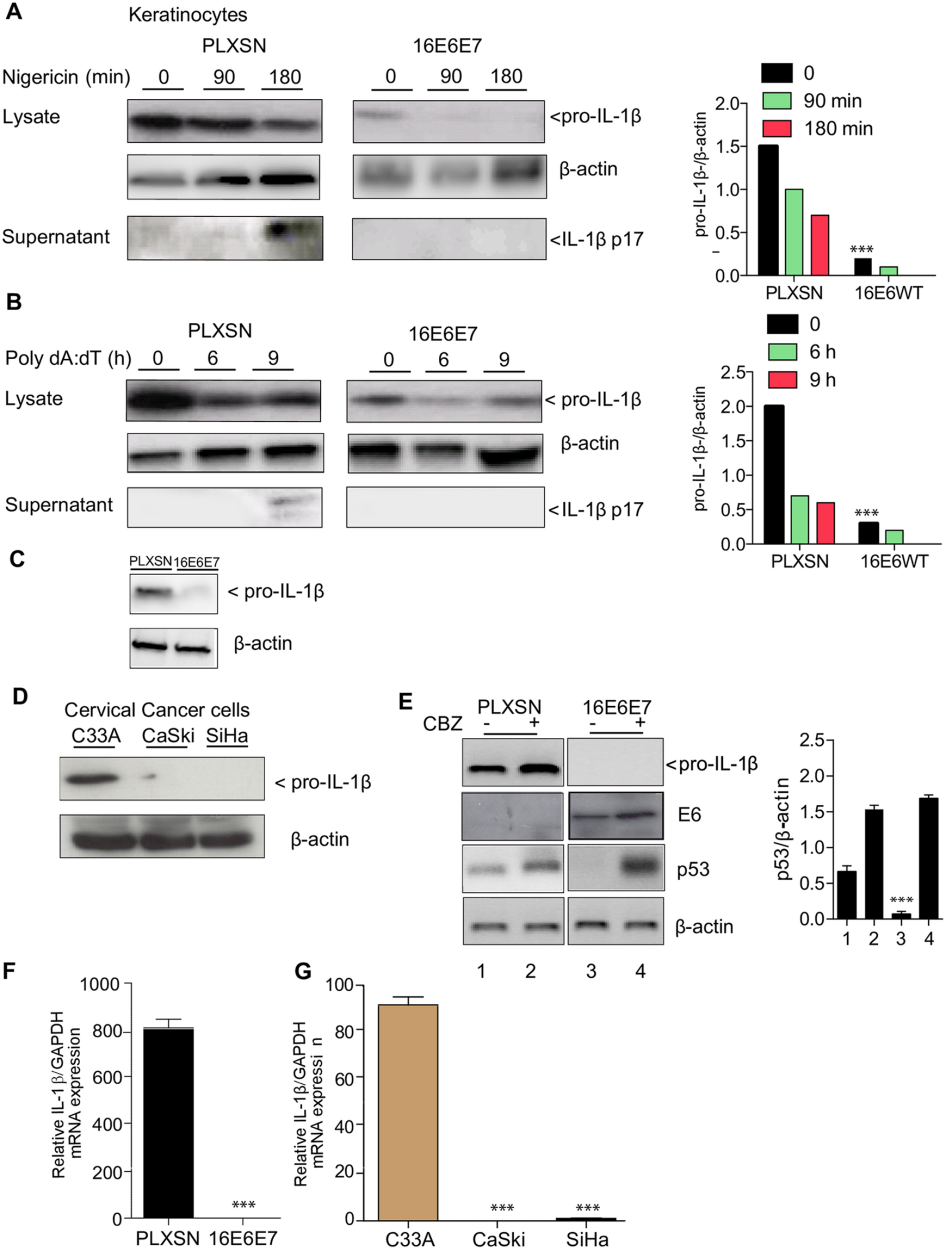
HPV16 oncoproteins inhibit pro-IL-1β levels. (A) Human keratinocytes transduced with pLXSN or 16E6E7 were stimulated with AIM2 and (B) NLPR3 ligands and both pro-IL-1β and IL-1β from cell lysates or supernatants were analysed by immunoblotting. β-actin was used as a loading control. Densitometry analysis was performed n = 3. (C) Immunoblotting of pro-IL-1β in human keratinocytes transduced with pLXSN or 16E6E7. (D) Cervical cancer cell lines were lysed and immunoblotting for pro-IL-1β was performed. n = 4 (E) Human keratinocytes transduced with pLXSN or 16E6E7 were treated for 24 h with N-CBZ-Leu-Leu-Leu-al. Cells were harvested and p53, E6 as well as pro-IL-1β levels were determined by immunoblotting. Right, p53 densitometry levels were normalized to β-actin. Below, immunoblot analysis of the 16E6 protein. n = 3. (F) RNA was extracted from Human keratinocytes transduced with pLXSN or 16E6E7 and IL-1β transcripts relative expression was determined by RT-qPCR. n = 5. (G) RNA was extracted from patient derived cervical cancer cell lines and IL-1β transcripts were determined by RT-qPCR. n = 6. Panels A-E. Data are representative of n independent experiments performed in triplicate. Shown are the mean ± SEM with ***, P < 0.0001, based on a two way ANOVA test. Panel F P < 0.0001, based on a one way ANOVA test. Panel G Student unpaired T test was performed comparing C33A to CaSki or SiHa. For immunoblotting data, 1 out of 3 experiments is shown.

We hypothesized that the loss of IL-1β production might be due to the ability of 16E6E7 to block NLPR3 and AIM2 transcription. Neither AIM2 nor NLRP3 transcript levels were altered in human primary keratinocytes transduced with 16E6E7, compared to the pLXSN control (S2A Fig). HPV16 E6 and E7 interact with p53 and retinoblastoma (pRb), respectively, and promote their degradation via the proteasome pathway [19]. Therefore, we next determined whether a similar mechanism affected NLRP3 or AIM2 protein expression in 16E6E7-expressing keratinocytes. Human NLRP3-CFP, AIM2-CFP or p53 constructs were co-transfected with 16E6E7 or pLXSN in human primary keratinocytes and their expression was examined by immunoblotting. We did not observe any alteration in AIM2 or NLRP3 protein levels. As expected we found that p53 was degraded by 16E6E7 (S2B Fig). As we did not detect any change at the receptor level, we next focused our attention on the downstream signaling molecules that are shared between NLRP3 and AIM2. Inflammasome activation requires ASC dependent caspase-1 maturation of pro-IL-1β [12]. Neither ASC nor caspase-1 transcript levels were altered in 16E6E7 compared to pLXSN transduced cells (S2C Fig). In addition cleavage of pro-caspase-1 was detected in 16E6E7 transduced cells stimulated with NLRP3 or AIM2 ligands (S2D Fig). We observed that levels of the pro-form of IL-1β were already reduced in 16E6E7 compared to LXSN transduced cells. These data indicated that the synthesis of IL-1β was affected by the viral oncoproteins before AIM2 or NLRP3 stimulation (Fig 3A–3C). The same loss of pro-IL-1β was observed in cervical cancer cell lines positive for HPV16 (Fig 3D). All these observations showed that 16E6E7 exerts an inhibitory effect on the synthesis of the pro-form of IL-1β. While Niebler et al., previously reported the ability of 16E6 to degrade pro-IL-1β via the proteasome [8], under our experimental conditions the addition of a specific proteasome inhibitor on 16E6E7 expressing keratinocytes did not restore the pro-IL-1β protein (Fig 3E). As expected, p53 levels increased in the presence of 16E6E7 confirming the specificity of the proteasome inhibitor (Fig 3E). Protein levels for 16E6 were controlled by western blot (Fig 3E). Indeed an alternative hypothesis was that 16E6E7 proteins can alter IL-1β mRNA, as shown by Karim et al, and Niebler et al., [7,8]. We observed that 16E6E7 blocked the level of IL-1β transcripts compared to normal cells (Fig 3F). Little or no IL-1β mRNA was detected in CaSki or SiHa compared to C33A cells (Fig 3G). These data indicated that 16E6E7 in human keratinocytes as well as in cervical cancer cells supresses mRNA expression of IL-1β.

### 16E6 as well as E6 from other high-risk HPV types block IL-1β transcription

**Fig 4:**
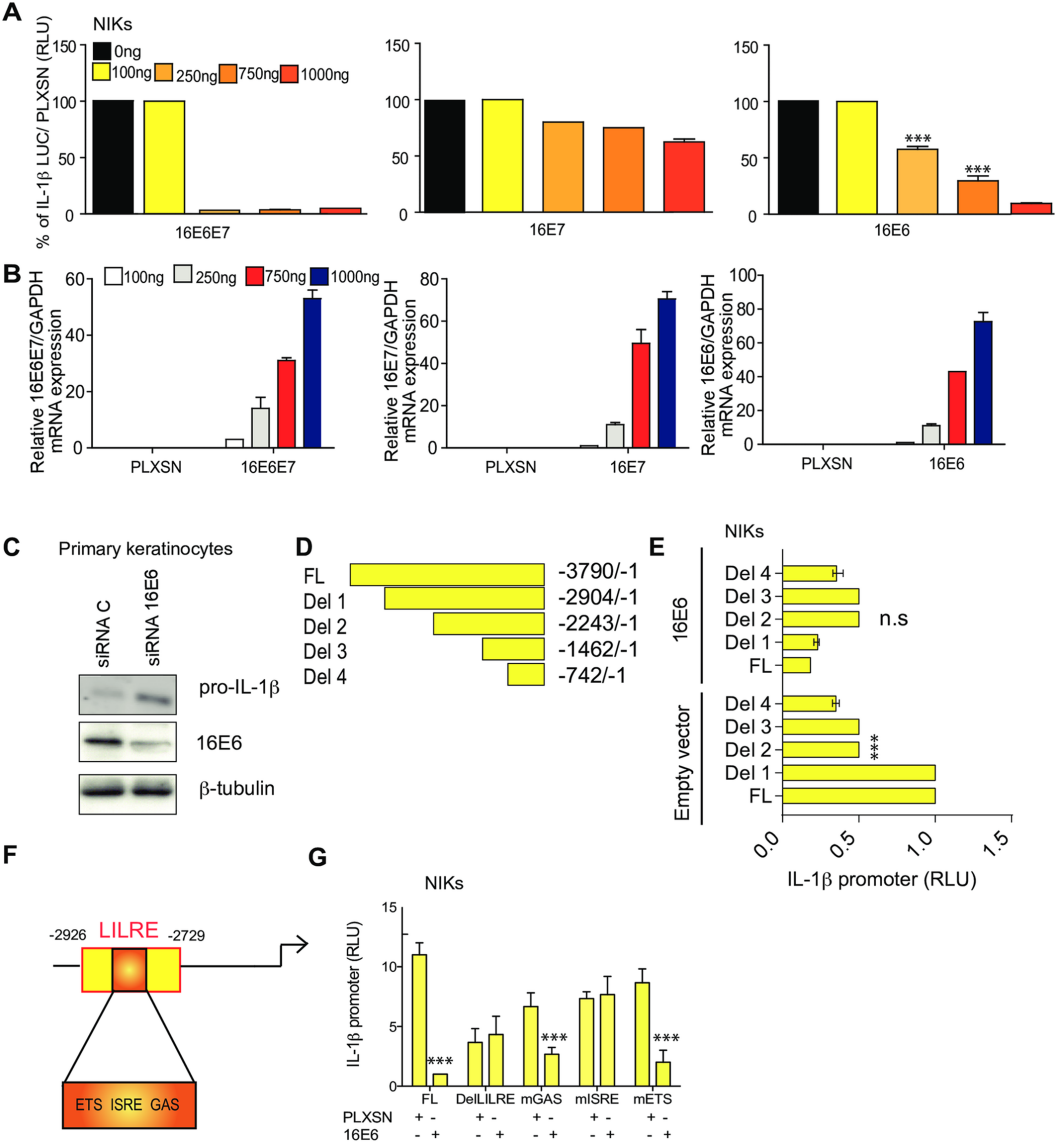
HPV16 E6 down-regulates the IL-1β promoter in cervical cells via the ISRE site. (A) NIKs were co-transfected with the IL-1β promoter with increasing concentrations of pLXSN HPV16E6E7 or 16E6 or 16E7 as indicated. After 48 h, cells were harvested and luciferase activity was measured. n = 5. (B) Relative expression of 16E6E7, 16E6 or 16E7 were measured by RT-qPCR. n = 5. (C) Primary human keratinocytes transduced with 16E6 and treated with a scramble or siRNA against 16E6. Protein levels of pro-IL-1β and loading control β-tubulin were evaluated by immunoblotting. n = 4. (D) Schematic representation of IL-1β promoter luciferase deletion mutations. (E) WT and deleted IL-1β promoter constructs were transiently transfected into NIKs expressing pLXSN or 16E6. After 48 h, cells were harvested and luciferase activity was measured. n = 4. (F) Schematic representation of the IL-1β LILRE site. (G) WT and deleted or mutated IL-1β promoter constructs were transiently transfected into NIKs expressing pLXSN or 16E6. After 48 h, cells were harvested and luciferase activity was measured. n = 4. Data are representative of n independent experiments performed in triplicate. Panel A and B shown are the mean ± SEM with ***, P < 0.0001, based on a two way ANOVA test. Panel F, is based on an one way ANOVA test and panel G a paired T test. For immunoblotting data, 1 out of 4 experiments is shown.

HPV16 may use E6 and/or E7 to directly inhibit IL-1β transcription. To determine whether HPV16 E6 or E7 proteins influence IL-1β transcription, the IL-1β promoter linked to the luciferase reporter gene was co-transfected ±16E6E7, 16E6 or E7 into spontaneously immortalized human keratinocytes (NIKs). NIKs already expressed high protein levels of endogenous pro-IL-1β. Indeed high basal luciferase activity was detected in these cells after transient transfection. However, 16E6E7 inhibited IL-1β luciferase activity even with low DNA concentrations (Fig 4A left), indicating that 16E6E7 can block the transcription of the IL-1β.. Furthermore 16E6, and to a lesser extent 16E7, inhibited IL-1β promoter activity (Fig 4A and 4B). Knock down of 16E6 restored pro-IL-1β (Fig 4C). We also compared the efficiency of E6 from other high-risk (HR) human papillomavirus types and one low risk (LR) type in repressing IL-1β transcriptional activity. HR types 18E6 and 31E6 inhibited IL-1β transcription, although less efficiently than 16E6 (S3A Fig). LR HPV6E6 did not affect IL-1β promoter activity (S3A Fig). These data demonstrated that E6 from HPV16 as well as other HR types strongly inhibit IL-1β transcription.

### The inhibition of IL-1β transcription by 16E6 involves an ISRE *cis* element on the IL-1β promoter

We next made deletions in the promoter to determine which region is required by 16E6 to inhibit IL-1β transcription. (Fig 4D). WT and IL-1β deletion constructs were co-transfected with 16E6. We restored IL-1β promoter activity with deletion 2 in the presence of 16E6 (Fig 4E). The deletion contains an area called LILRE was previously characterized by Unlu and colleagues [20]. The LILRE element has a high degree of inter-species conservation and plays and important role in IL-1β regulation (Fig 4F). Within the LILRE region, Unlu et al., showed the involvement of three different protein binding sites [20], an Spi-1 cis site (ETS); an IRF8-binding site (ISRE) and a Stat1 cis site (GAS) [20].

We hypothesized that 16E6 requires the regulatory LILRE site to inhibit IL-1β transcription. To test this, primary human keratinocytes were co-transfected ± 16E6 or pLXSN with WT, delLILRE (deletion of the LILRE site) and constructs that contained point mutations (m) for ISRE, ETS or GAS on the IL-1β promoter. Luciferase activity was restored with the delLILRE promoter indicating that this site contains a region required for IL-1β inhibition by 16E6 (Fig 4G). Luciferase activity remained suppressed in cells transfected with ETS mutant, suggesting that this *cis* element was not involved in the down-regulation of IL-1β transcription by 16E6 (Fig 4G). Luciferase activity was partially rescued in cells transfected with the mGAS promoter (Fig 4G). However, a complete rescue was observed in cells that were transfected with the mISRE promoter in the presence of 16E6. These results suggested that IL-1β down regulation by 16E6 principally involves the ISRE site on the IL-1β promoter.

### 16E6 expression inhibits the binding of IRF6 and not IRF8 on the IL-1β promoter

**Fig 5:**
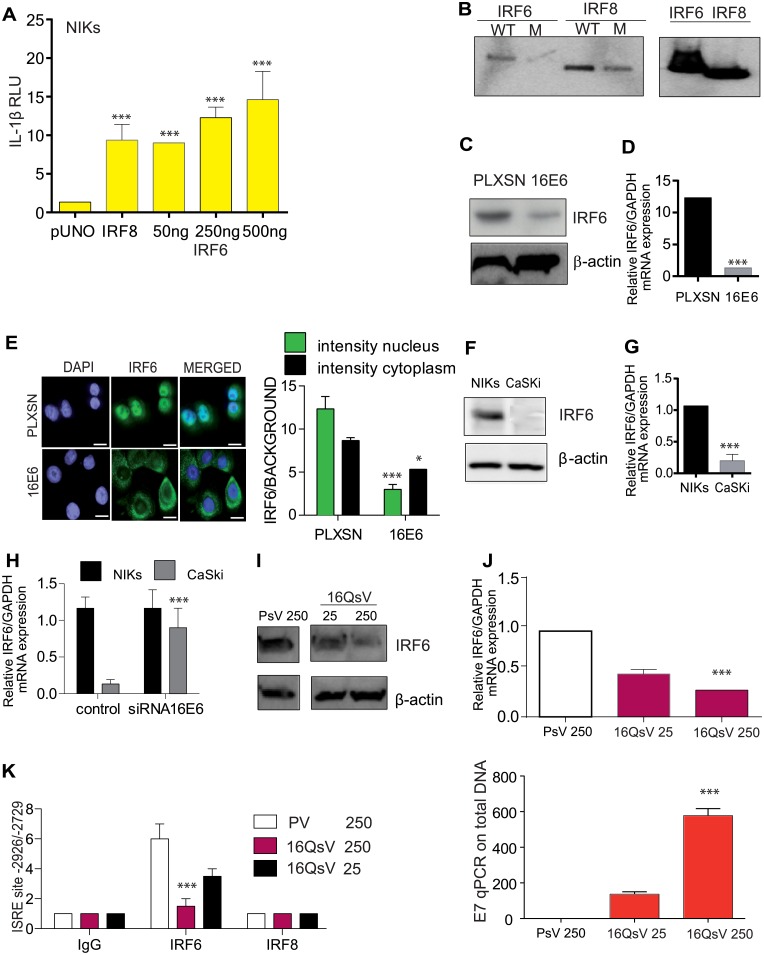
IRF6 and not IRF8 is recruited to the IL-1β promoter which is blocked by HPV16E6. (A) HEK293 cells were co-transfected with IL-1β promoter luciferase construct along with the empty vector pUNO, IRF8 or IRF6 plasmid at the indicated concentration. Post 48 h cells were lysed and luciferase activity measured. n = 4. (B) Oligo pulldown assay for WT or the mutated ISRE site using protein lysates from HEK293 cells transfected with IRF6 or IRF8. Bound proteins were assessed by immunoblotting for IRF8 or IRF6. Input controls (10%). n = 4. (C) Immunoblot analysis of IRF6 protein levels in in pLXSN and 16E6 transduced human primary keratinocytes. n = 4. (D) IRF6 relative levels were measured in pLXSN, 16E6 and 16E7 transduced human primary keratinocytes by RT-qPCR. n = 4. (E) Immunofluorescent staining of IRF6 in human keratinocytes transduced with pLXSN or HPV16E6. Left, semi-quantative analysis of IRF6 was examined by calculating immunofluorescent intensity. The mean and S.E.M of five fields were plotted. n = 4. (F) Immunoblot analysis of IRF6 protein levels in C33A and NIKs. n = 4. (G) IRF6 mRNA levels detected by RT-qPCR in NIKs and CaSki cells. n = 4. (H) NIKs and CaSki cells were co-transfected with IL-1β promoter luciferase construct ± siRNA for 16E6. Post 48 h cells were lysed and luciferase activity measured. (I) C33A cells were treated with control PsV or 16QsV at different v.g.e per cell for 24 h and IRF6 protein levels were examined by immunoblot. n = 3. (J) C33A cells were treated with control PsV or HPV16 at different v.g.e for 24h and IRF6 mRNA levels were examined by RT-qPCR and (below) viral DNA expression of E7 vs β2-microgloubulin. n = 3. (K), ChIP using IgG, IRF6 or IRF8 antibodies was performed for the ISRE site on C33A cells infected with HPV16 or PsV for 24 h. n = 3. Data are representative of n independent experiments performed in triplicate. Panels A, E, J and K are shown as the mean ± SEM with ***, P < 0.0001, * P, < 0,01, based on a two way ANOVA test. Panels D and G are shown as the mean ± SEM with ***, P < 0.0001 based on an unpaired T test. For immunoblotting data, 1 out of 4 experiments is shown. For immunoblotting data, 1 out of 4 experiments is shown.

The observation that an ISRE site is required for IL-1β suppression by 16E6 prompted us to determine which transcription is involved in this event. IRF8 is required for the development of monocytes, macrophages, dendritic cells (DCs), basophils, and eosinophils, while it inhibits the generation of neutrophils [21], yet nothing has been described for its role in keratinocytes. We observed no difference in gene or protein expression of IRF8 in primary human keratinocytes vs. 16E6 or E7 transduced cells (S3B Fig). Furthermore, by ChIP we observed in human macrophages IRF8 binding on the ISRE element, however in human keratinocytes we failed to demonstrate binding (S2C Fig). We concluded that IRF8 did not regulate the IL-1β promoter in human keratinocytes. In contrast to most IRFs, IRF6 has no identified function in innate immunity but is essential for normal keratinocyte epidermal development and differentiation [22]. We hypothesized that IRF6 might be involved in IL-1β transcription. To test this we co-transfected the IL-1β promoter with IRF8, IRF6 or pUNO expression vectors in HEK293 cells. As expected, IRF8 induced a significant increase in IL-1β luciferase activity when compared to pUNO transfected cells (Fig 5A). We also observed for the first time that IRF6 expression also increased IL-1β promoter activity in a dose dependent manner (Fig 5A). Oligo pull-down assays revealed IRF6 as well as IRF8 specific binding to the ISRE site on the IL-1β promoter (Fig 5B).

Having established that IRF6 binds to the IL-1β promoter and induces IL-1β transcription, we hypothesized that 16E6 might alter IRF6 expression. Indeed, IRF6 expression in human keratinocytes was decreased in cells expressing 16E6 (Fig 5C and 5D). Furthermore, immunofluorescence detection of IRF6 in primary keratinocytes was localized in the nucleus but shifted into the cytoplasm in 16E6 cells (Fig 5E). ImageJ analysis of IRF6 fluorescence showed that both cytoplasmic and nuclear levels were reduced in keratinocytes expressing 16E6 (Fig 5E). Furthermore, both mRNA and protein levels for IRF6 were lower in CaSki (HPV16+) versus NIKs (Fig 5F and 5G). siRNA targeting of 16E6 reversed the effect, and IRF6 levels were resorted (Fig 5H). We also observed that IRF6 protein levels and mRNA levels were reduced when epithelial cells were treated with increasing amounts of 16QsV (Fig 5I and 5J). The decrease of IRF6 mRNA levels was inversely proportional to viral DNA expression of E7 (Fig 5J). ChIP assays revealed that IRF6 bound less to the ISRE element when cells were infected with 16QsV (Fig 5K).

In summary, we confirmed that IRF8 is required to induce IL-1β expression in monocytes, yet in human keratinocytes IRF6 regulates IL-1β transcription. Furthermore, IRF6 binding to the ISRE site on the IL-1β promoter is inhibited by 16E6 expression in primary human keratinocytes.

### 16E6 mutations reveal that E6 degradation of p53 is required to inhibit IRF6 transcription

**Fig 6:**
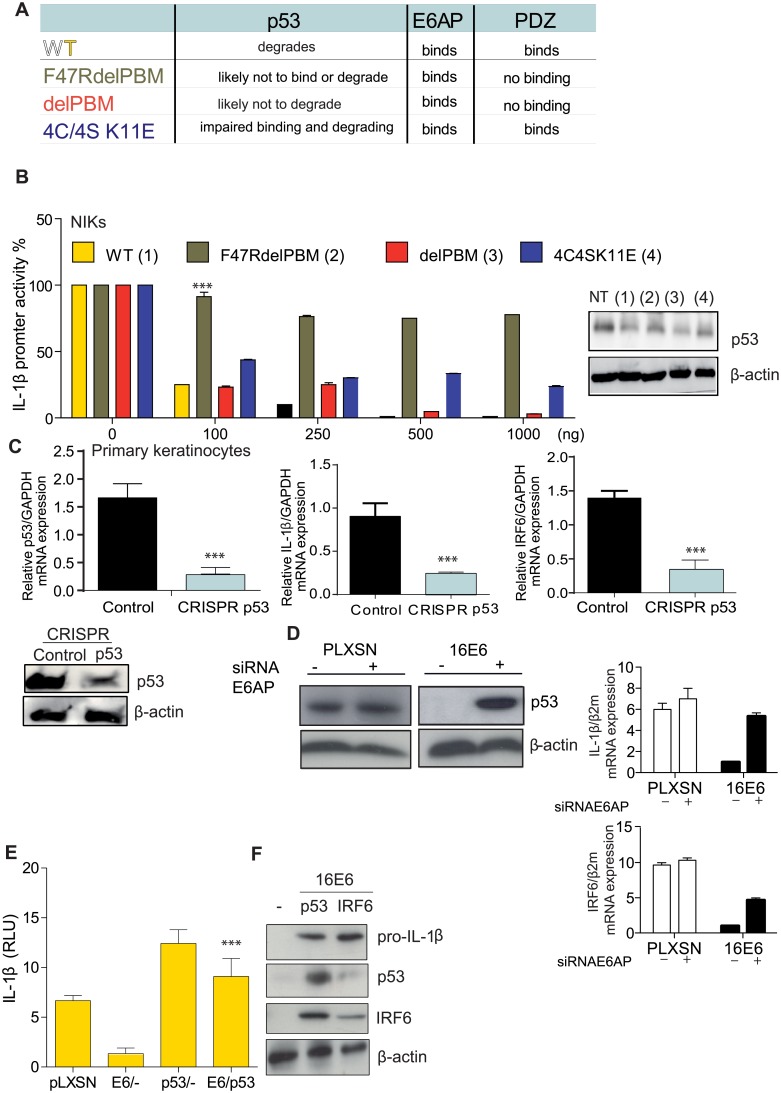
Loss of p53 inhibition by 16E6 restores IRF6 activity. (A) Table defining the 16E6 mutations that alter p53, E6AP or PDZ binding sites. (B) NIKs were co-transfected with IL-1β promoter luciferase construct along with the HPV16E6 WT and mutated constructs at the indicated concentration. n = 5. (C) Human primary keratinocytes were transfected with cas9/sgRNA for p53 or control and 36h later cells were examined for mRNA levels for p53, IL-1β or IRF6. n = 4. (D) siRNAE6AP (+) or siRNA scramble control (-) was transfected into PLXSN or 16E6 transduced human keratinocytes for 48 h, cells were harvested for protein and RNA. Western blot analysis for p53 and β-actin. Left top, RT-qPCR for IL-1β and, left below IRF6. n = 3, (E) NIKs were co-transfected with the IL-1β promoter and pLXSN, E6, p53 or E6 with p53. Luciferase activity was measured 48 h post infection. n = 4. (F) 16E6 transduced primary keratinocytes were transfected with vector control (-), p53 or IRF6 expression vectors. Twenty-four hours later cells were harvested and pro-IL-1β, p53 or IRF6 levels were examined by immunoblotting. n = 4. Data are representative of n independent experiments performed in triplicate. Shown are the mean ± SEM with ***, P < 0.0001, based on an one or two way (applicable to > 2 conditions) ANOVA test. For immunoblotting data, 1 out of 4 experiments is shown.

The HPV16 oncoprotein E6 interacts with numerous proteins by hijacking several host cellular networks. To gain further insight into the mechanistic role of 16E6 on IL-1β transcription, we co-transfected the IL-1β promoter with plasmid constructs that contain point mutations that alter E6 binding to cellular host proteins [23,24,25,26,27,28] (S4A and S4B Fig and Fig 6A). We then co-transfected increasing amounts of 16E6 WT or mutations with the IL-1β promoter (Fig 6A and 6B). IL-1β luciferase activity was restored with the 16E6F47RdelPBM mutant and partial restored with delPBM and 4C/4S K11E. These data indicated that the 16E6F47R mutation, which fully disrupts its ability to degrade p53 [23,28] can no longer block IL-1β transcription. These data suggest that p53 also regulates IL-1β transcription (Fig 6A and 6B). We next explored the role of p53 on IL-1β transcription. We suppressed p53 expression in primary human keratinocytes using the CRISPR/CAS9 technology (Fig 6C). Suppression of p53 led to a decrease in IL-1β and IRF6 transcription (Fig 6C). Blocking 16E6 mediated E6AP proteosome degradation of p53 using a siRNA for E6AP restored p53 protein levels as well as IL-1β and IRF6 mRNA expression (S4C Fig and Fig 6D). Over expression of p53 restored IL-1β promoter activity in the presence of 16E6 (Fig 6E). In addition, overexpression of p53 or IRF6 expression in keratinocytes transduced with 16E6 also reconstituted pro-IL-1β protein levels (Fig 6E). Taken together, these data show that 16E6 degradation of p53 is required to inhibit IL-1β transcription.

**Fig 7:**
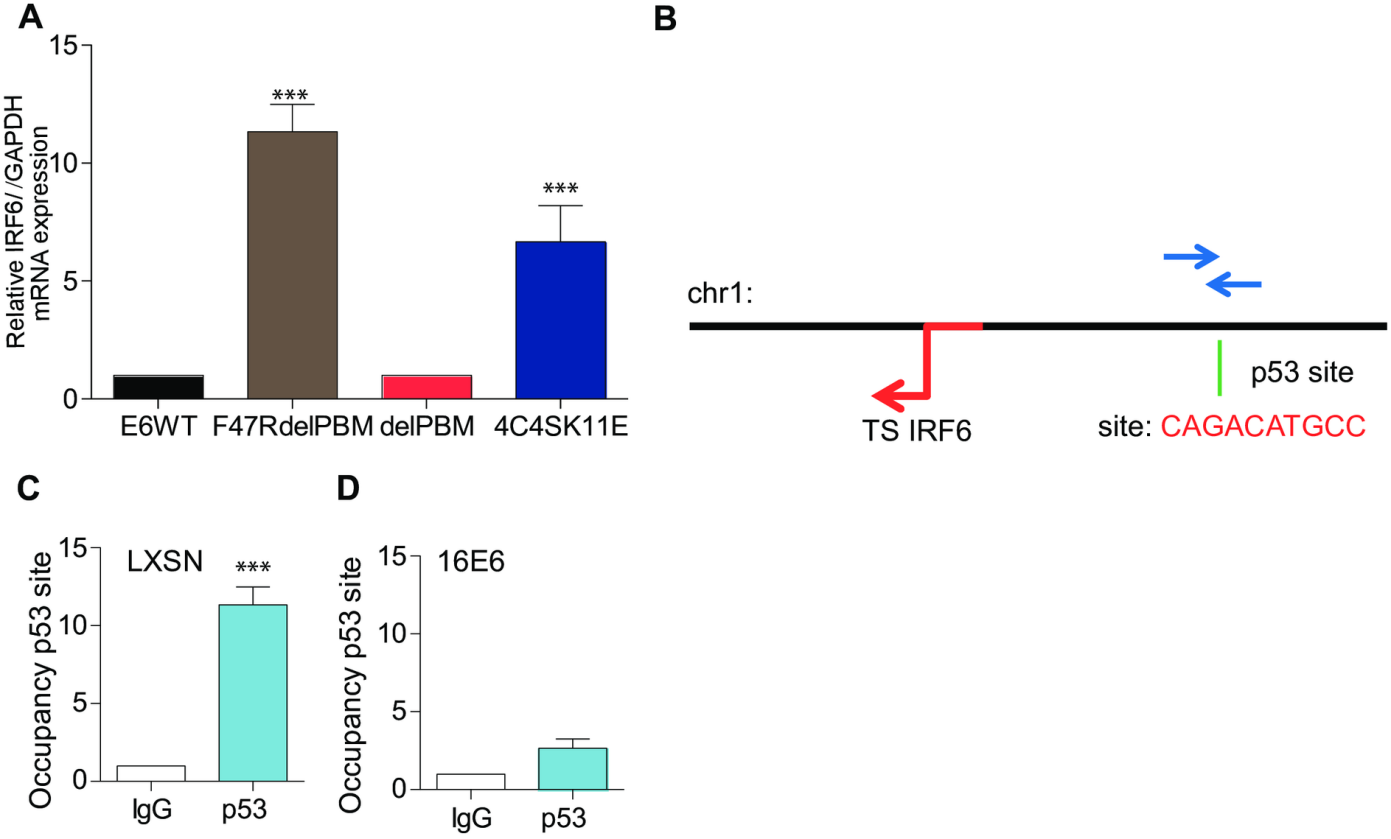
p53 is a transcription factor required for IRF6 transcription in human keratinocytes. (A) HPV16E6WT or mutations were transfected into NIKs and IRF6 mRNA levels were measured by RT-qPCR. Data were normalised to β2-microglpbulin and GAPDH house-keeping genes. n = 4. (B) Schematic diagram of the p53 binding site and sequence on the IRF6 promoter. The red arrow indicates the transcription start site along Chr1. The green line indicated where the p53 cis element is located (written in red). The blue arrows indicate the primer amplication over 200bp. (C, D) ChIP assay of p53 binding on the IRF6 promoter in human primary cells (LXSN) as well as in 16E6 transduced cells. n = 3. Data are the representative of n independent experiments performed in triplicate. Shown are the mean ± SEM with ***, P < 0.0001, based on an one way ANOVA test.

So far we have shown that both IRF6 and/or p53 regulate IL-1β transcription and that both proteins are blocked by 16E6. Whether both proteins independently or dependently control IL-1β transcription remained to be determined. IRF6 transcription was no longer inhibited when cells transiently expressed 16E6 mutations that altered p53 degradation (Fig 7A). Based on these data we hypothesized that p53 regulates IRF6 transcription. Indeed, using the gene card software, we identified a *p53* cis element on the IRF6 promoter. We, therefore, performed ChIP experiments in human primary keratinocytes ±16E6 to determine if p53 was able to bind to the IRF6 promoter (Fig 7B). We observed that p53 bound to the *cis* element on the IRF6 promoter in human keratinocytes (Fig 7C and 7D). Occupation of this site was reduced in 16E6 expressing cells (Fig 7C and 7D). In summary we demonstrated the existence of a negative feedback loop in which 16E6 degradation of p53 prevented the transcription of IRF6 and the subsequent transcription of IL-1β.

### IRF6 transcriptional regulation by p53 is lost in cervical neoplasia

**Fig 8:**
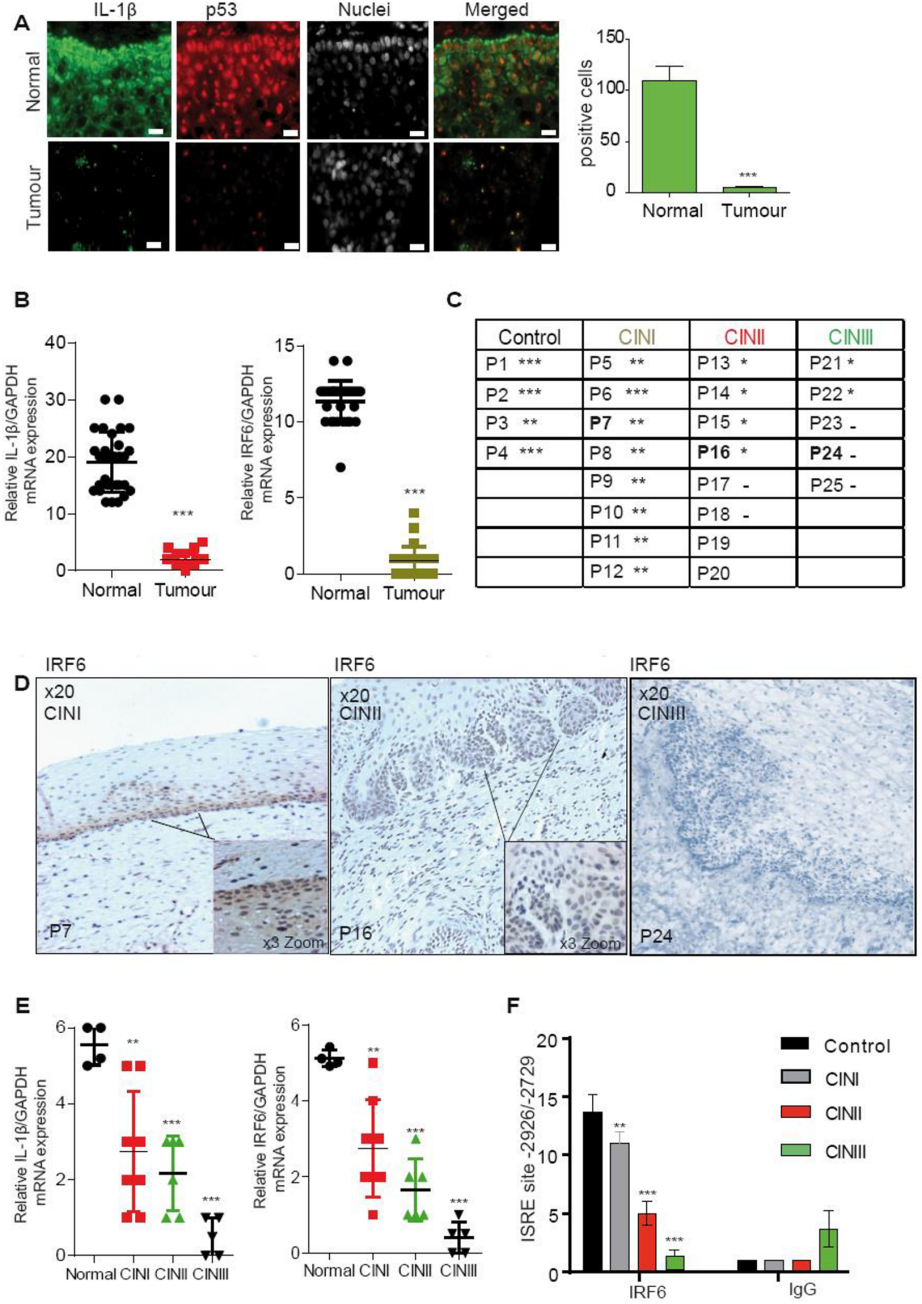
HPV16-positive cervical cancer lesions contain less IRF6 and IL-1β. (A) Immunofluorescence of normal cervical issue and HPV16+ cervical cancer biopsies. IL-1β (green), p53 (red) with trace indicating the basal layer and nucleus (white). Normal (HPV−) and a neoplastic biopsy (HPV16+) from one representative patient out of six with similar results are shown. Bars represent a scale of 10 μm. For each stained biopsy, six fields were examined IL-1β staining was counted manually and the percentage scored out of 100 cells. n = 4. (B) RNA was extracted from normal (29) and cervical cancer biopsies (29). IL-1β relative and IRF6 mRNA levels were measured by RT-qPCR. n = 4. (C) Table of immunohistochemical scoring IRF6 in patients at different stages of cervical neoplasia. Scoring, *** strong, ** medium, * low and—no staining (4 normal, 8 CINI, 8 CINII, 5 CINIII). n = 2. All tissue staining data were examined by two pathologists. (D), Immunohistochemical staining of IRF6 in cervical tissue in patients with CINI, II or III. n = 2. (E) RT-qPCR of IL-1β and IRF6 mRNA expression levels in normal vs neoplastic cervical tissue. n = 3. **A p53 site is required to bind to the IRF6 promoter but is lost in cervical cancer tissues**. (F) ChIP analysis was performed on normal and cervical neoplastic tissue for IRF6 binding on ISRE site on the IL-1β promoter. n = 3.

Our next approach was to validate our *in vitro* findings in patients with cervical cancer. (HPV16 +). Cervical cancer and matched normal tissue biopsies were taken from 6 patients and snap frozen. After analysis and HPV typing, sections were stained by immunofluorescence for IL-1β as well as p53. Basal cells of the normal epidermis showed strong cytoplasmic staining for IL-1β and nuclear staining for p53 (Fig 8A). No staining for IL-1β and p53 was observed in tumour cells (representative staining in Fig 8A). Quantification of the cytoplasmic staining clearly showed that IL-1β expression was strongly down-regulated in cancerous compared to normal tissue (Fig 8A). We next wanted to determine if IL-1β and IRF6 transcripts were down regulated in cervical cancer patients. To do this we used a larger cohort from normal (29 patients) and cervical tumor biopsies (29 patients) and RNA was extracted. RT-qPCR of IRF6 and IL-1β transcripts revealed that both genes were reduced in tumor tissues compared to normal biopsies (Fig 8B).

**Fig 9:**
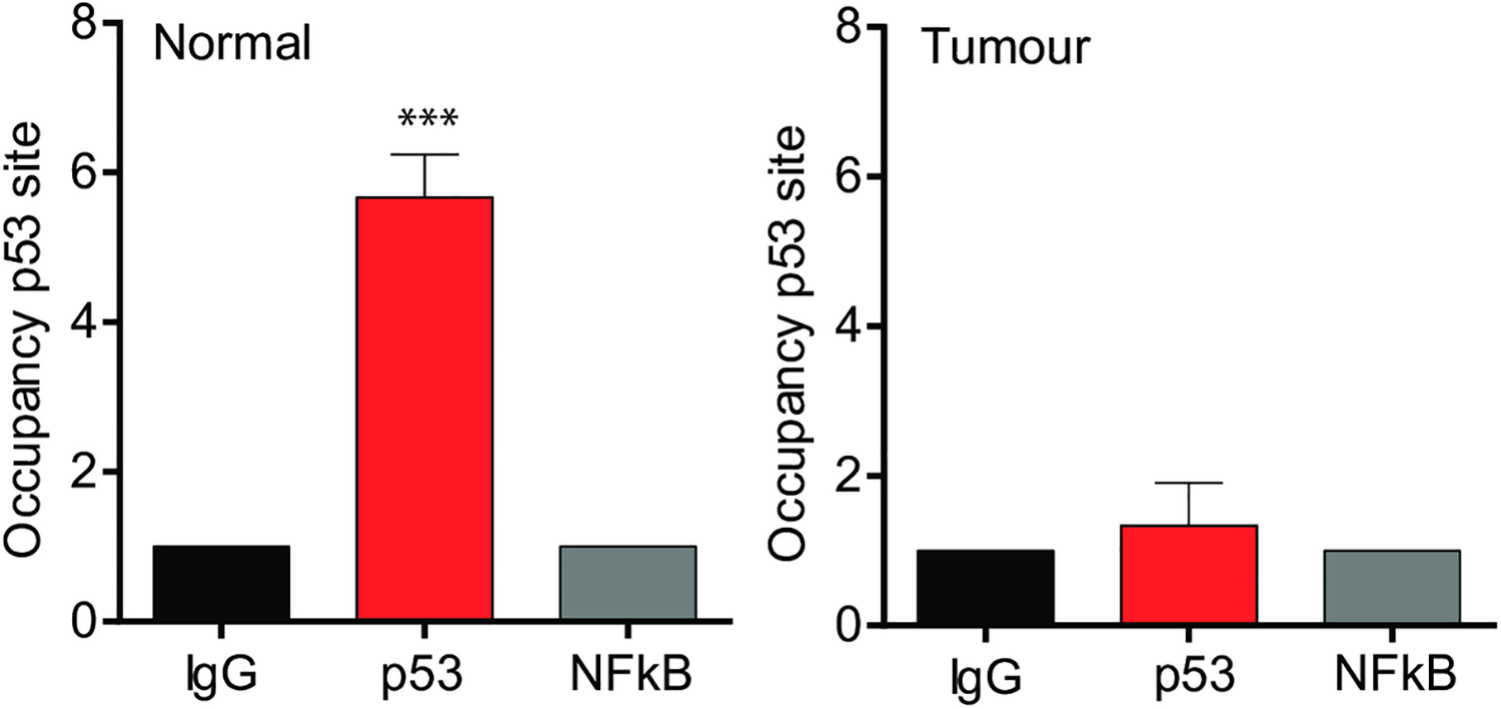
Four normal and tumor biopsies from (A) were used to perform ChIP analysis for p53 binding onto the IRF6 promoter. n = 3. Data are representative of n independent experiments performed in triplicate. Shown are the mean ± SEM with ***, P < 0.0001, **, P < 0.0005 based on a paired Student’s t test (when patient matched) or unpaired when not patient matched.

Cervical intraepithelial neoplasia (CIN) is the premalignant abnormal growth of squamous cells on the surface of the cervix. Most cases of CIN remain stable, or are eliminated by the host’s immune system without intervention. We next explored if IL-1β and IRF6 transcription were altered in patients during the progression of CIN positive for HPV16. We obtained Formalin-Fixed Paraffin-Embedded biopsies from normal cervical tissues (n = 4) as well as HPV16-positive CINI (n = 8), II (n = 8) and III (n = 5; Fig 8C). Immunohistochemical staining of normal cervical tissue revealed high nuclear expression of IRF6 in the basal layers; which decreased as CIN status increased (Fig 8C and 8D). We observed a decrease in both IRF6 and IL-1β mRNA during disease progression, (Fig 8E). ChIP experiments using chromatin extracted from patient tissue revealed that IRF6 binding to the ISRE site on the IL-1β promoter decreased during CIN severity (Fig 8F), indicating that loss of IRF6 inversely correlates with cervical neoplasia progression. Furthermore, p53 binding was observed in normal cervical biopsies, but binding was reduced in patients with cervical tumors (Fig 9). These data strongly suggest that the p53/IRF6 regulation of IL-1β transcription is lost during CIN disease stages that could lead to cervical cancer.

## Discussion

**Fig 10:**
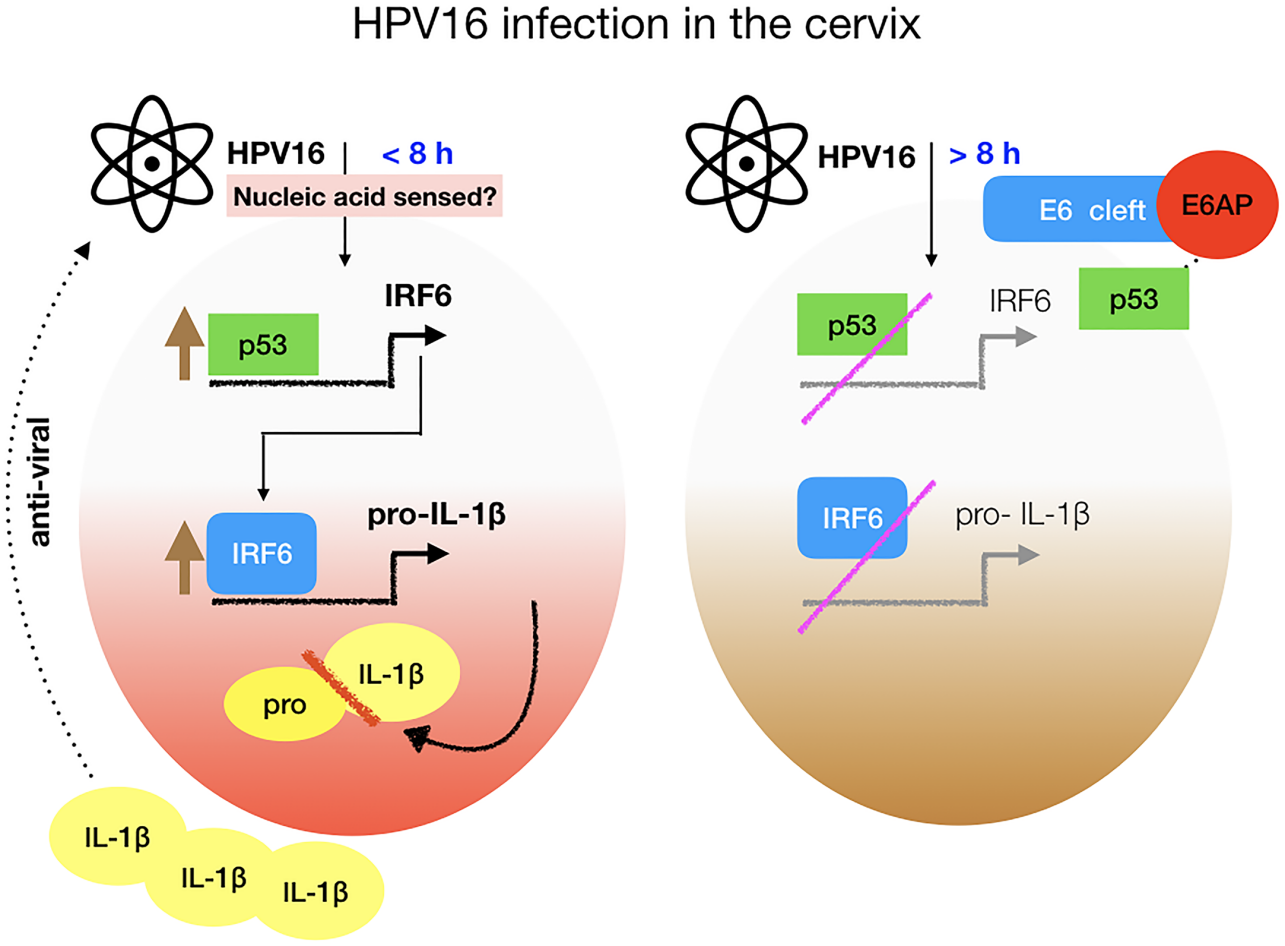
Model representing the induction and inhibition of IL-1β by HPV16. Infection of the basal keratinocytes with HPV16 induces inflammasome dependent IL-1β production sensed by an unknown innate receptor. p53 transcriptional regulation of IRF6 is increased, which we show drives IL-1β transcription. The pro form of IL-1β is cleaved by caspase 1 (red bar). The active form of IL-1β can block the increase in viral copies. However, when the oncoprotein E6 is expressed this drives p53 degradation by E6AP preventing IRF6 and consequently IL-1β transcription. This mechanism of viral inhibition of innate responses may contribute to HPV16 persistence in the host.

We showed that human keratinocytes produce IL-1β when exposed to 16QsV. Furthermore, addition of recombinant IL-1β on 16QsV infected keratinocytes led to a block in viral gene transcription. Viral gene transcription was restored in the presence of an antagonist for the IL-1 receptor. More importantly we delineated that IL-1β gene transcription increased when exposed to 16QsV. These data show that HPV16 stimulates IL-1β secretion that has an anti-viral effect on infected cells. IL-1β depends on inflammasome activation; we have data showing that 16QsV was not sensed by NLPR3 or AIM2 (S5A Fig). Bone marrow derived macrophages from NLRP3 and AIM2 knock out mice were still able to produce IL-1β in the presence of 16QsV (S5A Fig). Therefore we still need to elucidate which innate-inflammasome sensor can detect 16QsV. However IL-1β gene expression began to decrease post 8h infection with 16QsV. These data implicate that HPV16 has developed an escape mechanism to block IL-1β production. Our findings are summarised in Fig 10.

Characterizing how HPV blocks immune surveillance is central in understanding the events involved in the establishment of head and neck as well as cervical cancers. In this study we showed the loss of IL-1β transcription was mediated mainly by oncoprotein 16E6. Our data are in line with Karim et al., showing that IL-1β mRNA levels were decreased in epithelial cells expressing 16E6E7 [29]. The addition of 16QsV, or expression of E6 alone, blocked IRF6 expression and binding to the ISRE site on the IL-1β promoter. IRF6 has previously been shown to play an important role in the embryonal development of the craniofacial region. Mutations in this gene have been found in two human syndromes: Van der Woude and Popliteal Pterygium Syndrome, which are characterized by the cleft palate, lip pits, skin webbings, syndactyly, genital deformities and oral adhesions. In contrast to most IRFs shown to be essential in IFN gene regulation, IRF6 had no identified function in innate immune gene activation. Other IRF family members have been shown to be hijacked during HPV mediated carcinogenesis, such as IRF1 [30]. We demonstrated that mutation of the ISRE site on the IL-1β promoter prevented 16E6 to inhibit IL-1β promotor activity. Gene silencing of the viral oncoproteins 16E6E7 or 16E6, restored IRF6 and IL-1β expression in human keratinocytes. This was shown by calculating the percentage of 16E6 inhibition against the cells that are induced with the PLXSN vector alone (S5B Fig). Furthermore we showed that p53 regulated IRF6 transcription.

Using 16E6 mutations that cannot, partially or fully degrade p53 allowed us to correlate the degradation of p53 by 16E6 led to the loss of IRF6 transcription (Fig 10). p53 has also been shown to amplify intracellular IFN responses. IFN-stimulated genes (ISG) promoters do not contain p53 consensus binding sites. However Munoz-Fontela et al., identified IFN regulatory factor 9 (IRF9), a component of the ISG factor 3 (ISGF3) complex, as a p53 target gene. ISGF3 directly induces the expression of ISRE-containing genes and could represent a mechanistic link between p53 and ISG induction [31]. Several additional IFN-stimulated mediators of ISG expression, including IFN regulatory factor 5 (IRF5), immune-stimulated gene 15 (ISG15) and the Toll-like receptor 3 (TLR3), have been identified as direct p53 target genes. Therefore IRFs and p53 play a central role in regulating innate immune responses.

To our knowledge, this is the first description of p53-IRF6 axis mediating differential regulation of an immune gene. Our ChIP experiments showed that lack of p53 protein due to 16E6 prevented its recruitment to the IRF6 promoter in cervical cancer patients. Based on our findings we hypothesized that loss of IRF6 and IL-1β expression favours cervical cancer development. These data were corroborated in cervical neoplasia and tumours. In cohorts of cervical neoplastic patients we observed a decrease in both IL-1β and IRF6 mRNA levels. Rotondo et al., evaluated the gene expression changes involved in neoplastic progression of cervical intraepithelial neoplasia compared to normal keratinocytes [32]. Microarray analysis revealed that IRF6 was one of the 24 genes significantly down regulated during CIN progression [32]. Furthermore two independent studies showed that IRF6 gene mutations were associated to head and neck squamous cell carcinomas [33] [34]. However these scientific findings conflict with two other data sets. Our analysis of the data set by den Boon et al., showed that IL-1β was not affected during cervical cancer progression [35]. Also neither IRF6 nor IL-1β mRNA levels were suppressed when analysing the data set from the TCGA cervical carcinoma cohort [36]. One should consider that neither studies were hypothesis driven nor were the data sets designed to examine the mechanism of HPV16E6 regulation on p53/IRF6/IL-1β. We validated that IRF6 and IL-1β expression were altered by the viral oncoprotein 16E6 using several read-outs and models. Furthermore, we showed that IL-8 gene transcription depends on IL-1β stimulation. An increase in local cervical IL-8 levels correlates with HPV viral clearance [37]. Experts in HPV incidence have discussed that infection of the cervical epithelium is a prerequisite for the development of cervical cancer and the local immune response is an important determinant of progression and disease outcome [38]. The transiency of most HPV infections and the observed regression of certain cervical intraepithelial neoplasia lesions to normal epithelium suggest a change in local immune responses, which may be caused by differences in host genomics. We observed that loss of IL-1β production in cervical cancer cells led to a loss of paracrine IL-8 transcription. Furthermore, IL-1β down regulation in HPV induced carcinogenesis is underlined by the fact that specific polymorphisms in IL-1β have been demonstrated to be associated with cervical carcinoma risk [38].

The work of Niebler et al., showed that 16E6 alters IL-1β by proteosome degradation of the pro-form [8]. We did not observe the same findings using our cellular models. This could be due to the fact that the primary keratinocytes used by Niebler et al., were from neonatal foreskin, whereas our model used keratinocytes from adult female skin (see Method and materials). Yet, Niebler et al., also showed in Fig 6 of their article a drop in mRNA IL-1β levels in CIN patients [8]. These data fall in line with our findings.

We propose that inflammasome activation of IL-1β secretion favors’ HPV viral clearance. Loss of IRF6 and IL-1β function during cervical neoplastic stages reflects a prognostic read out towards cancer development. Thus, interfering with the regulation of IL-1β with synthetic agonists that target p53 and IRF6 levels may provide a novel therapeutic strategy for cervical cancer patients.

## Methods and materials

### Cell culture

Cervical cancer cell lines C33A (HPV negative cat: HTB-31), SiHa (HPV16 positive cat: HTB-35), CaSki (HPV16 positive cat: CRL-1550), HeLa (HPV18 positive cat: CCL-2) and Human embryonic kidney 293 (HEK293 cat: CRL-1573) cells were purchased from American Type Culture Collection (Manassas, VA) and cultured in DMEM medium (Life technologies), supplemented with 10% foetal bovine serum (FBS), L-glutamine, pyruvate and 0.1% ciprofloxacin (Euromedex). HEK293TT cells were a kind gift from the lab of Dr. Pawlita (DKFZ, Germany). Cells were cultured with hygromycin using the same culture medium as HEK293. When preparing HEK293TT cells for transfection cells were grown without hygromycin and antibiotics. Cells were cultured at 37°C with 5% CO_2_. Immortalized near-diploid human keratinocyte cell line (NIKS, kind gift from Professor John Doorbar, University of Cambridge, UK) and Human Primary Keratinocytes produced by the lab of Massimo Tommasino were from Adult female, or femaile skin keraintocytes were purchased from American Type Culture Collection Cat: PCS-200-011). Cells were cultured as previously described [6]. Human Primary Keratinocytes were cultivated at low passages numbers for a period of 3 weeks (called keratinocytes after 1 passage). High-titer retroviral supernatants (>5 × 10^6^ IU/ml) were generated as previously described [39]. The 16QsV and PV production, infection, and viral genome expression quantification of HPV16 are described below.

### Agonists and antagonists

NLRP3 ligand Nigericin was used at 1μg/mL (Sigma), AIM2 ligand poly(dA:dT) was used at 1μg/well (Invivogen) and transfected using lipofectamine 2000 (Invitrogen). ANAKINRA (Biovitrum) was used at 200μg/ml.

### Oligo pull-down

Oligo pulldown was performed as previously described [40] with cellular extracts as stated in the figure legend and oligo probes as listed in Table 1. IRF8 and IRF6 antibodies were purchased from Cell Signaling.

**Table 1:** Oligo sequences.

*Deletions IL-1β*	*OLIGO PULL DOWN IL-1β*
FWD 1 CTAGCTAGCTCTAGACCAGGGA	FWD Biotin’ TTTGACATAAGAGGTTTCACTTCC
FWD 2 CTAGCTAGCTAAGAGGTTTCACT	REV GGAAGTGAAACCTCTTATGTCAAA
FWD 3 CTAGCTAGCCTCCAGCCTGGGG	*IRF6 qPCR*
FWD 4 CTAGCTAGCCCTGAATGTACATGCC	FWD GGCATAGCCCTCAACAAGAA
FWD 5 CTAGCTAGCTTAGGCAGAGCTCAT	REV CACCCCTTCCTGGTACTTCC
REV 1 GAAGATCTAAGAGGTTGGTA	*IRF8 qPCR*
REV 2 GAAGATCTAAGAGGTTTG	FWD ACGAGGTTACGCTGTGCTTT
	REV GACATCTCGGCAGGGCTATG
	*p53 qPCR*
	FWD GGTTTGTAATGCAGGGCTGAGG
	REV GGGTATGGTGGTGTATGCCTGT

### ChIP

ChIP assays were performed using the Shearing Optimization kit and the OneDay ChIP kit (Diagenode). For C33A cells or primary keratinocytes, cell sonication cycles last 15s with 5s on and 2 s off at 20% of amplitude and were repeated four times. For tissue, immunoprecipitation was performed overnight on a rotating wheel at 4°C. 2.5 μl/reaction of DNA solution was used for qPCR. The primers used to amplify IL-1β, or IRF6 binding regions are available on request. ChIP on the tissue was performed according to the protocol from Epigenome Network of Excellence for tissue preparation after the Red ChIP kit from diagenode was used to prepare chromatin and the 1-d ChIP kit for the immunoprecipitation. Immunoprecipitation was performed overnight on a rotating wheel at 4°C. 2.5 μl/reaction of DNA solution was used for qPCR.

### Plasmid constructs

The constructs pLXSN empty, pLXSN-16E6E7, pLXSN-HPV16E6, pLXSN-HPV16E7 and pLXSN-HPV18E6E7 were obtained from M.Tommasino (IARC, Lyon, France) (6). The pGL3 Luc vector was purchased from Promega. The constructs The full-length IL-1β-Luc, LILRE (IL-1 response element) and mutants were obtained from Philip E.Auron (University of Pittsburgh, Pittsburgh, PA 15261, USA). IL-1β deletions were cloned using the primers listed in Table 1. Nine E6 mutations were obtained from Dr Gilles Trave (CNRS, Illkirch, France); and previously described. These mutations were cloned into the pX5 plasmid. The retroviral pBabe-puro encoding HPV16 and 6 E6 and or E7 have been previously described [41]. The constructs pLXSN-HPV16 E6, HPV18 and HPV38 E6 and HPV6 E6 were a gift from D. Galloway (Fred Hutchinson Cancer Research Center, Seattle, WA). The plasmids used for HPV16 structural genes and control PsV production, the target HPV16 genome, and GFP (for PsV control) were kindly donated from the laboratories of Martin Muller and Angel Alonso (DKFZ, Germany). pUNO, human IRF6 and IRF8 constructs were purchased from Invivogen. The p53 plasmid was obtained from Addgene. siRNA for 16E6E7 and E6 was purchased from Dharmacon and Sigma respectively. siRNA for E6AP [42]CRISPR for p53 was purchased from Santa Cruz.

### Viral production 16QsV and PsV [43],[44]

16QsV are viral particles that contain the full viral genome of HPV16 encaspidated by the viral late proteins L1 and L2. PsV contain GFP DNA encaspidated by L1 and L2.

293TT cells at 75% confluency the day of transfection. The transfection mix consisted of 13μg of the L1-L2 expression vector and ~ the same amount of HPV16DNA or GFP control vector were prepared in a separate tube, a mix 85μl of Lipofectamine with 2ml OptiMEM. Both mixtures were incubated separately at RT for 10´-30´, then combined and incubated for at least another 20 minutes. The resulting lipid/DNA complexes were directly added to the pre-plated cells. The cells were incubated with the transfection mix for 4–6 h then split 1:2 or 1:3 and left overnight. The next day cells were detached, spun down and the supernatant discarded. *Cell lysis and Capsid Maturation*: Using a 5ml plastic pipet, cells were suspended in 0.5ml in DPBS-Mg and transferred to a siliconized 2ml tube, screw-capped (Nalgene tubes for freezing cells). For 100 million cells 1 ml of lysis buffer was prepared and incubated for 1-2h at 37C then with inversion for a further 16 h at least at 37C.

### Salt extraction

The next day optiprep gradients prepared were diffused for 4 hours. The lysate was then layered on top of the gradient. The tubes were spun for using 13.2ml tubes SW40.1 Ti 14 h at 16 C. The L1 band is a visible as a slight grey layer a little over a third of the gradient. Using a large needle and a 5ml syringe we removed the 60% cushion layer, then we tool a 1.0ml syringe and 26 gauge needle to extract 250μl fractions (6–8 fractions). Each fraction was placed into a screw cap tube (not freezing tubes). *Screen fractions by SDS PAGE*: A mini gel of 10% were used to screen fractions for the presence of the L1 protein (55kDa) fractions with significant amounts of L1 were pooled, aliquoted and frozen; the protein yield can be estimated through BSA standards or BCA assay. *Analysis of virions- Encapsidated DNA*: Fifty μl of fractions were run on a 0,8% agarose gel. Supercoiled DNA from the HPV genome, linear human DNA with nucleases and exonuclease treatment captured by L1 and L2 will run at 8Kb. *nuclease should cut up all the human genomic DNA, then any tailed DNA that gets incorporated into the capsid were cut off with the exonucleases. *Capsid protein levels*: Capsid protein levels (20μl fractions) were measured on 10% SDS-PAGE and silver staining with serially diluted BSA as concentration standard or by western blotting for L1. Viral genome equivalents were measured by qPCR on the viral DNA of infected HEK293T cells using W-12 cell lysates as a standard (kind gift from Dr Franck. Stubenrauch, Forschungssektion Experimentelle Virologie, Tubingen, Germany).

### Ethics statement

Our cohort of normal, CIN and tumor samples was provided by the hospital in Lyon Sud, Lyon, France. Samples were obtained with written informed consent from each patient with the procedure approved by the local Ethics Committee, Comités de Protection des Personnes. All, normal, CIN or tumor biopsies were from females aged between 30–50 years. Where available the same normal patient-matched samples were provided (HPV negative genotyped using multiplex PCR with HPV type-specific primers). Biopsies were either snap frozen or FPPE.

### Genotyping

CIN and Tumor samples were genotyped using multiplex PCR with HPV type-specific primers for amplification of viral DNA and array primer extension for typing [41].

### Infectivity and viral gene transcription assay

NIKs or primary keratinocytes were infected with packaged viruses as stated in the figure legends at 37C. Cells were removed, and RNA extracted for RT-PCR for E1, E6 and E7 transcripts (mRNA) or DNA to measure viral DNA expression for E7 [6].

### Immunofluorescence and immunohistochemistry

Keratinocytes transduced with pLXSN or HPV16E6 were fixed as previously described [45]. Sections of 5-μm thickness were cut and either stained for immunofluorescence using the TSA system (PerkinElmer). The p53 antibody was purchased from Cell Signaling and the anti-IL1β 3ZD (kindly provided by Dr. Trinchieri, NCI). The IRF6 antibody (F12) was purchased from Santa Cruz. Cells or tissues were washed, the coverslips were mounted onto slides using a 1/10 dilution of 4′,6′-diamidino-2-phenylindole (nuclear stain; Invitrogen) in fluoromount (Southern Biotechnology Associates), and protein expression was detected by direct fluorescence microscopy. Photographs were taken at magnification x40 using the Zeiss confocal 710 microscope. Semi-quantitative analysis of IRF6 levels was estimated using the ImageJ software. Immunohistochemistry staining for IRF6 was performed as previously described [6].

### ELISA

NIK, primary keratinocytes, HPV16E6 and E7 induced keratinocytes were seeded into a six-well plate with 2.5 x10^5^ cells per well with 4x10^3^ NIH 3T3 feeders. Two days later the feeders were removed, and the medium was replaced. After two hours; keratinocytes were stimulated either with 20μM of Nigericin (Sigma) or transfected with 1ug/mL of poly (dA:dT) (Invivogen) using lipofectamine 2000 (Invitrogen). After the indicated period (see figure legend), the supernatant was harvested and quantified for IL-1β by ELISA (Bender Med System) or IL-18 [46].

### Luciferase assay

Twenty-four hours before transfection HEK293 cells were plated at 20% of confluency in 96 well plates with 180μl of complete medium per well. Cells were transfected using GeneJuice Transfection Reagent (Novagen) following the manufacturer’s instructions. Cells were transiently co-transfected with HPV constructs as indicated with pGL3-LILRE, mutants or pGL3-XTLuc. A Renilla plasmid with a CMV promoter was used to normalize transfection efficiency. Twenty-four hours after transfection cells were lysed at room temperature in passive lysis buffer (Promega) for 20 minutes. Luciferase buffer was composed of MgSO4 (2,67mM), EDTA pH8 (0.1 mM), DTT (33.3 mM), ATP (0.53 mM), acetyl-CoA (207 μg/ml), luciferin (0.13 mg/ml), Magnesium carbonate hydroxide (0,265 mM) and tricine (20 mM). Renilla buffer was made by diluting coelenterazine. Luciferase and renilla activity from transfected cells were measured using a luminoskan Ascent (Thermo). A single read program with an integration time of 1000 ms was used. Firefly luciferase (*Photinuspyralis*) activity of individual cell lysates was normalized against renilla (*Renillareniformis*) activity to correct for transfection efficiency in each reaction.

### IL-8 bioassay

Supernatants from stimulated cells were added onto HEK 293 cells transfected with IL-8 luciferase promoter, and a Renilla plasmid with a CMV promoter was used to normalise transfection efficiency [47]. Twenty-four post stimulation cells were processed as listed above.

### Protein/RNA extraction

Cells were preserved in RP1 lysis buffer complemented with β-mercaptoethanol (1%) until RNA and total proteins extraction using the NucleoSpin RNA/protein extraction kit (Macherey-Nagel). Supernatants from stimulated cells where concentrated using MeOH/chloroform. All RNA samples were treated with DNAse before reverse transcription was performed.

### Western blot analysis

Eighteen μg of total cellular protein were incubated during 5 minutes at 95°C. The protein samples were separated by electrophoresis using Novex 4–20% Tris-Glycine gels (Life Technologies) for 1 hour at 100V. Proteins then were transferred on a PVDF membrane (PerkinElmer) during 1 hour at 100V. After blocking with PBS 0.1% tween and 5% milk for 1 hour, membranes were probed with the following primary antibodies: anti-caspase 1 P10 (SantaCruz Biotechnology), anti-IL1β 3ZD (kindly provided by Dr Trinchieri, NCI), anti-ASC (Santa Cruz Biotechnology), 16E6 (provided by the lab of Dr Trave (GBMC, France) and 16E7 (Santa Cruz, France) over night at 4°C. β-actin (Sigma) primary antibodies were added for 2h at RT. After three PBS 0.1% tween washes, secondary antibodies are added for two hours at RT. Anti-Rabbit and anti-mouse HRP conjugate secondary antibodies were provided by Promega. Proteins were revealed with Lumiglo chemiluminescent substrate system (Kpl). Western blots were developed using the intelligent dark box (Fuji film).

### RT qPCR

We retro transcribed (RT) 1–1.5 μg of RNA extracted from cells using first strand RT-PCR kit with oligodT primers (Fermentas). The RT reaction was diluted according to detection sensitivity. One μl of the diluted samples was added to a 20 μl PCR mixture containing 0.4 μl of primers forward and reverse (10 μM) and 10 μl of Master Mix. Mx300P real-time PCR system (Stratagene, La Jolla, CA) were used to performed qPCR with Mesa Green qPCR Master Mix Plus (Eurogentec) on CaSki, C33A and SiHa cells. Primer sequences designed to detect gene expression of AIM2, NLRP3, ASC, IL-1β, house-keeping β2-microglubulin and GADPDH are listed as previously described [46]. As relative levels of house-keeping genes between samples did not alter, data were plotted against GAPDH. Primers for IRF6, IRF8, and p53 are listed in Table 1.

### Statisical tests

Where appropriate, anova, unpaired or paired T test were performed using prism software version 6 (Graph Pad) Statistical studies were validated by Omran Allatif (Statistician CIRI, Lyon, France[46,48]).

## Supporting information

S1 Fig IL-1β production by primary human keratinocytes. A: IL-1β was measured by ELISA in human keratinocytes (pLXSN) in response to NLRP3 or AIM2 ligands. n = 10. B IL-1β was measured by ELISA in human keratinocytes in response to PV or 16QsV at 250 v.g.e ± Glybride (inhibits ATP mediated proton pump) or ± Caspase-1 inhibitor. C IL-1β was measured a 4h and 8h by ELISA in human keratinocytes (pLXSN) in response to NLRP3, AIM2 ligands or 16QsV (left Y axis) or LDH release (right Y axis) using the Pierce ^™^ LDH kit (Thermofisher). n = 4. Shown are the mean ± SEM with ***, P < 0.0001, based on a two way ANOVA test.
(TIF)

S2 Fig 16E6E7 has no effect on the inflammasome activation of caspase-1. (A) RNA was extracted from Human keratinocytes ± 16E6E7 and NLRP3 or AIM2 relative expression was determined by RT-qPCR. n = 5. (B) Immunoblot analysis of keratinocytes transduced with LXSN or 16E6E7 were transfected with NLRP3-CFP or AIM2-CFP. Membranes were probed for GFP, p53 or β-actin n = 5. (C) RNA was extracted from human keratinocytes ± 16E6E7 and ASC or caspase-1 relative expression was determined by RT-qPCR. n = 5. (D) Human keratinocytes ± HPV16E6E7 were stimulated with AIM2 and NLPR3 ligands and both pro-or mature caspase-1 were analysed in cell lysates or in the supernatant by immunoblotting. β-actin was used as a loading control. n = 3.
(TIF)

S3 Fig Other HPV HR types but not LR blocks IL-1β promoter activity. (A) NIKs were co-transfected with the IL-1β promoter with pLXSN, 16E6, 18E6, 31E6 or 6E6 as indicated. After 48 h, cells were harvested and luciferase activity was measured. n = 5. **IRF8 is not involved in IL-1β transcription in human keratinocytes**. (B) IRF8 relative levels were measured in pLXSN, 16E6 and 16E7 transduced human primary keratinocytes by RT-qPCR. n = 4. Immunoblot analysis of IRF8 protein levels in in pLXSN, 16E6 and 16E7 transduced human primary keratinocytes. n = 4. (C) ChIP assay of IRF8 binding on the IL-1β promoter in human primary cells (LXSN) as well as in human macrophages. n = 4.
(TIF)

S4 Fig Mutations in 16E6 restore IL-1β activity. (A) Table describing 16E6 mutations. NIKs were transfected with 16E6Wt and mutations were co-transfected with IL-1β promoter luciferase construct. Forty-eight hours post transfection cells were lysed and luciferase activity measured. n = 4. (B) NIKs were transfected with WT and mutations for 16E6. Forty-eight hours post transfection proteins were probed using 16E6 antibody. n = 3. (C) Western blot to control E6AP knock down by control and SiRNA E6AP, using β-actin as a loading control. n = 4. Data are representative of n independent experiments; graphs shown are the mean ± SEM from triplicate values.
(TIF)

S5 Fig (A) 16QsV activates IL-1β production independently of AIM2 and NLRP3. Bone marrow derived macrophages from C56BL/6 WT, AIM2-/-, ASC -/-, Caspase 1 -/- (from Thomas Henry, France) and NLRP3 mice (From Virginie Petrilli, France) were isolated and cultivated as previously described [49]. (B) Percentage of IL-1β promoter inhibition with PLXSN cells vs 16E6 transfected with the IL-1β point mutation or LILRE deletion.
(TIF)
