# Supplementary figures and images for: Human papillomavirus type 16 antagonizes IRF6 regulation of IL-1β
Source: PLoS Pathog. 2018 Aug 8;14(8):e1007158. doi: 10.1371/journal.ppat.1007158 (PMC6124776; doi:10.1371/journal.ppat.1007158)

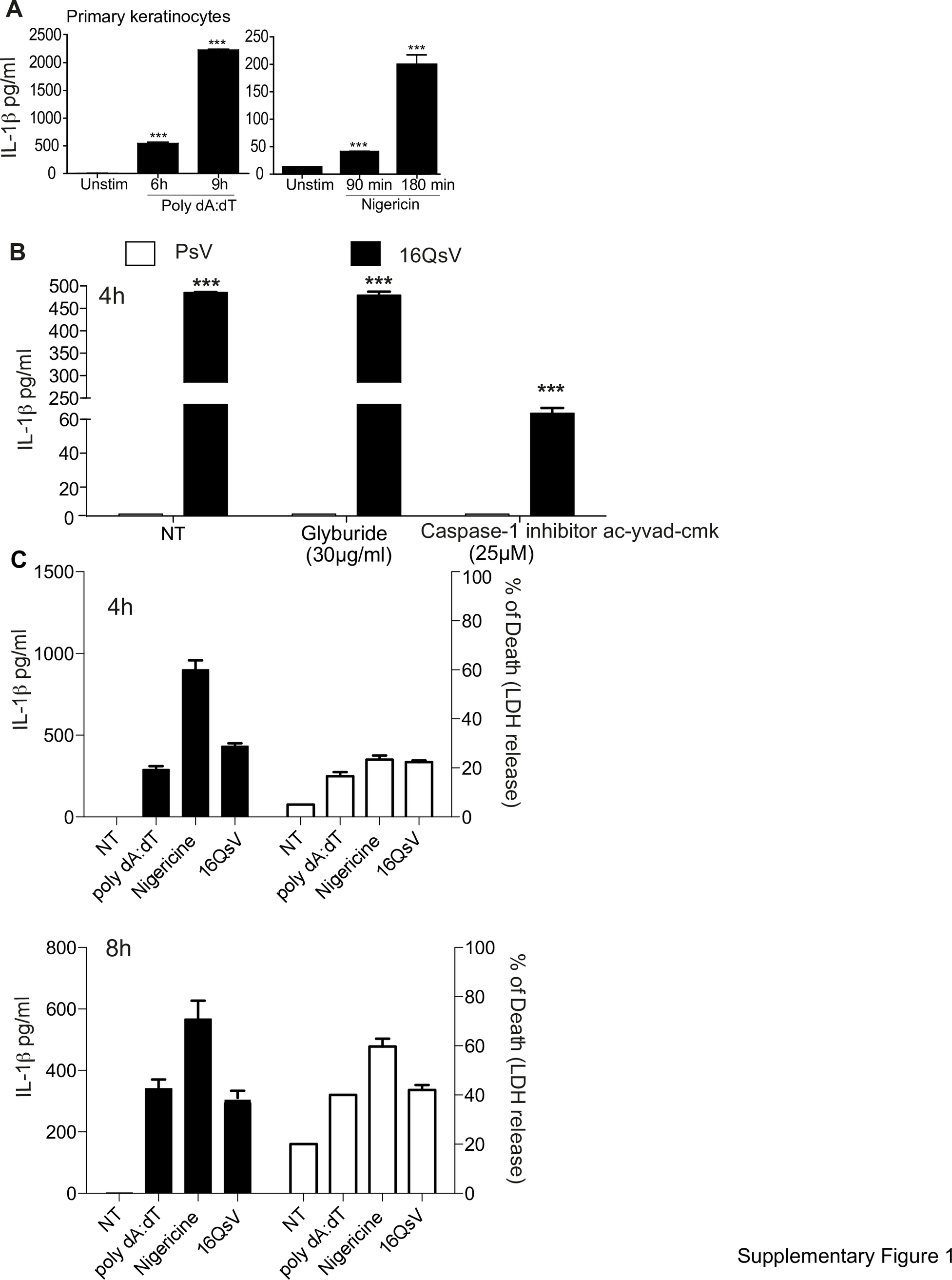

Supplement: S1 Fig — A: IL-1β was measured by ELISA in human keratinocytes (pLXSN) in response to NLRP3 or AIM2 ligands. n = 10. B IL-1β was measured by ELISA in human keratinocytes in response to PV or 16QsV at 250 v.g.e ± Glybride (inhibits ATP mediated proton pump) or ± Caspase-1 inhibitor. C IL-1β was measured a 4h and 8h by ELISA in human keratinocytes (pLXSN) in response to NLRP3, AIM2 ligands or 16QsV (left Y axis) or LDH release (right Y axis) using the Pierce ™ LDH kit (Thermofisher). n = 4. Shown are the mean ± SEM with ***, P < 0.0001, based on a two way ANOVA test. (TIF) [file ppat.1007158.s001.tif]

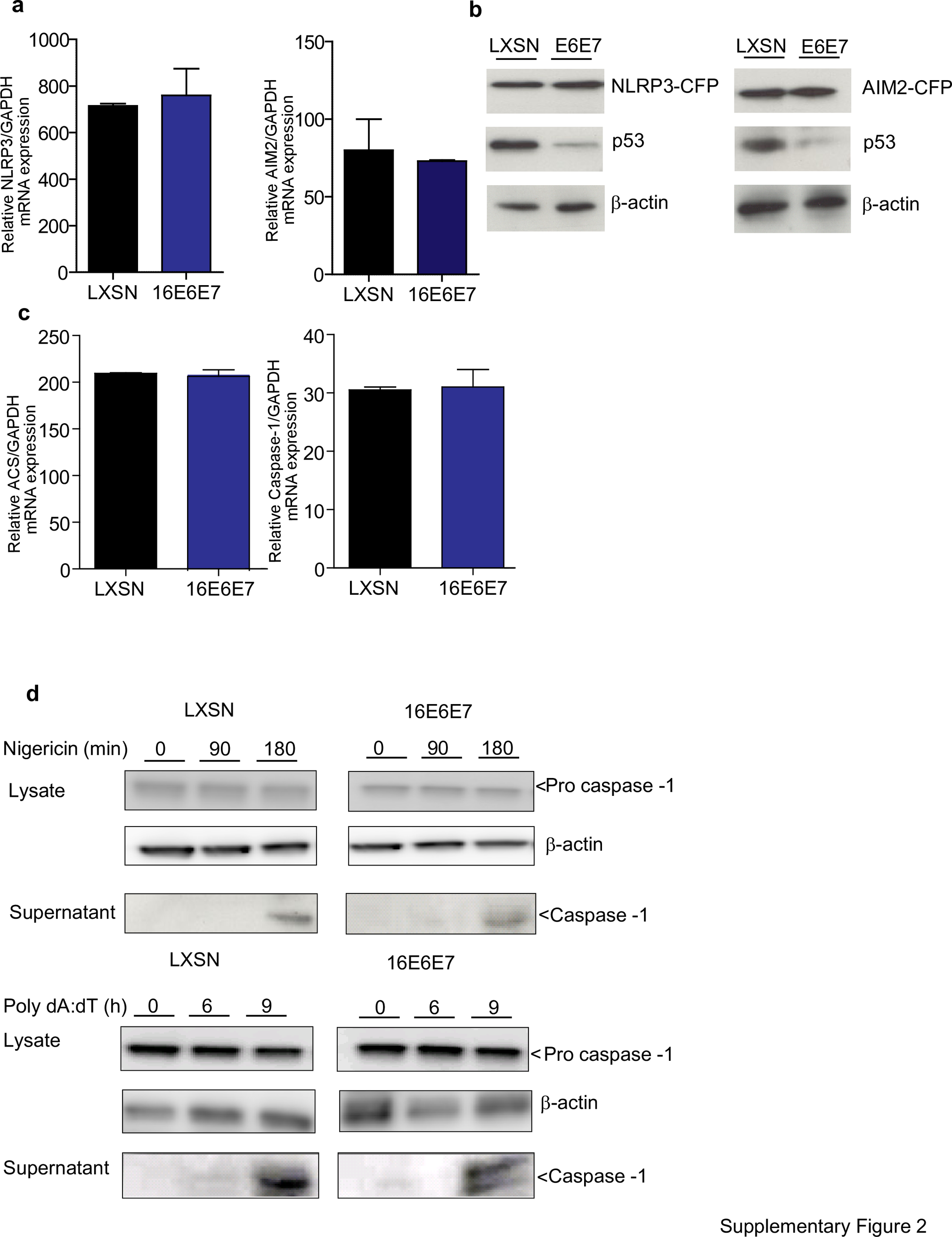

Supplement: S2 Fig — (A) RNA was extracted from Human keratinocytes ± 16E6E7 and NLRP3 or AIM2 relative expression was determined by RT-qPCR. n = 5. (B) Immunoblot analysis of keratinocytes transduced with LXSN or 16E6E7 were transfected with NLRP3-CFP or AIM2-CFP. Membranes were probed for GFP, p53 or β-actin n = 5. (C) RNA was extracted from human keratinocytes ± 16E6E7 and ASC or caspase-1 relative expression was determined by RT-qPCR. n = 5. (D) Human keratinocytes ± HPV16E6E7 were stimulated with AIM2 and NLPR3 ligands and both pro-or mature caspase-1 were analysed in cell lysates or in the supernatant by immunoblotting. β-actin was used as a loading control. n = 3. (TIF) [file ppat.1007158.s002.tif]

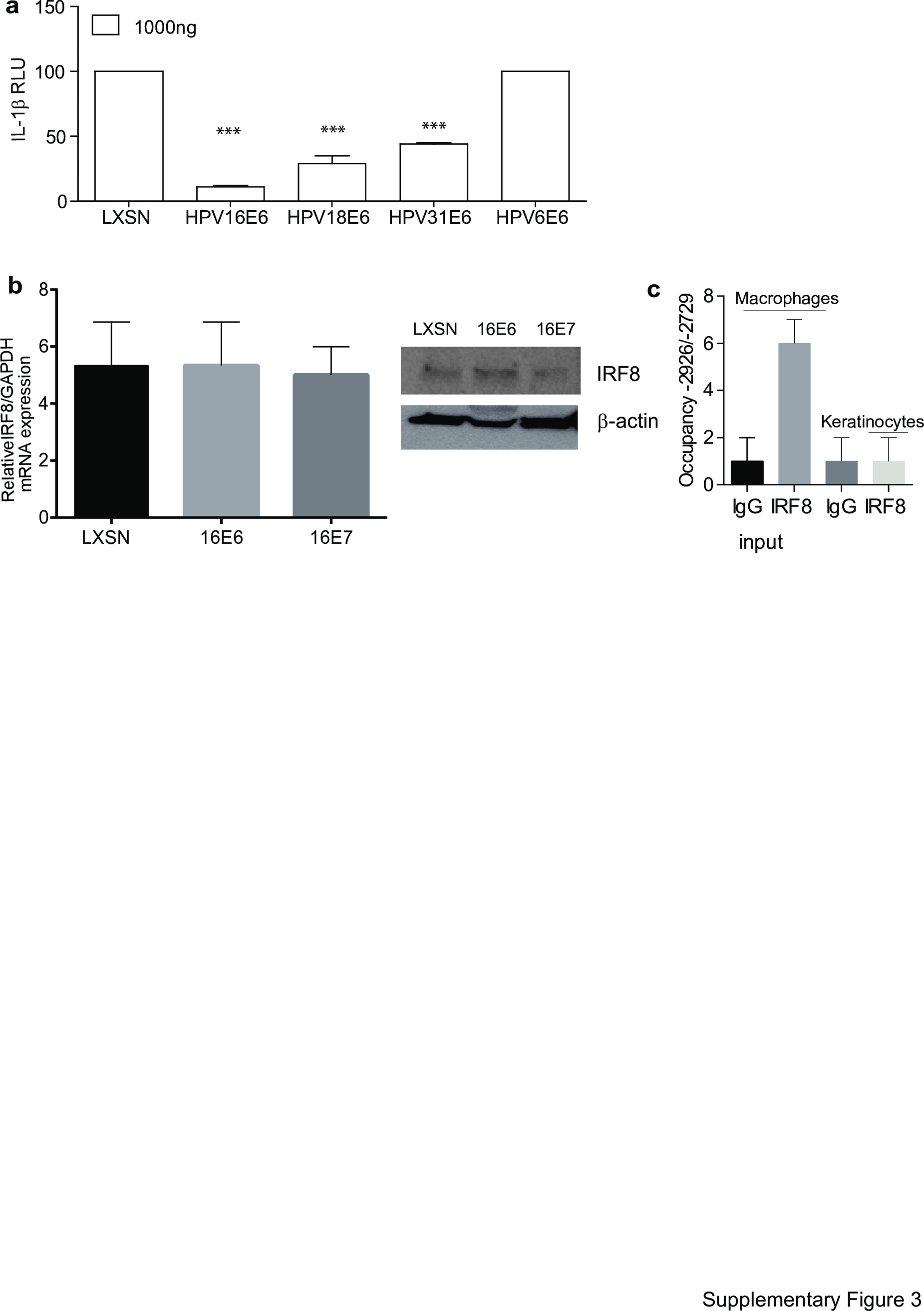

Supplement: S3 Fig — (A) NIKs were co-transfected with the IL-1β promoter with pLXSN, 16E6, 18E6, 31E6 or 6E6 as indicated. After 48 h, cells were harvested and luciferase activity was measured. n = 5. IRF8 is not involved in IL-1β transcription in human keratinocytes. (B) IRF8 relative levels were measured in pLXSN, 16E6 and 16E7 transduced human primary keratinocytes by RT-qPCR. n = 4. Immunoblot analysis of IRF8 protein levels in in pLXSN, 16E6 and 16E7 transduced human primary keratinocytes. n = 4. (C) ChIP assay of IRF8 binding on the IL-1β promoter in human primary cells (LXSN) as well as in human macrophages. n = 4. (TIF) [file ppat.1007158.s003.tif]

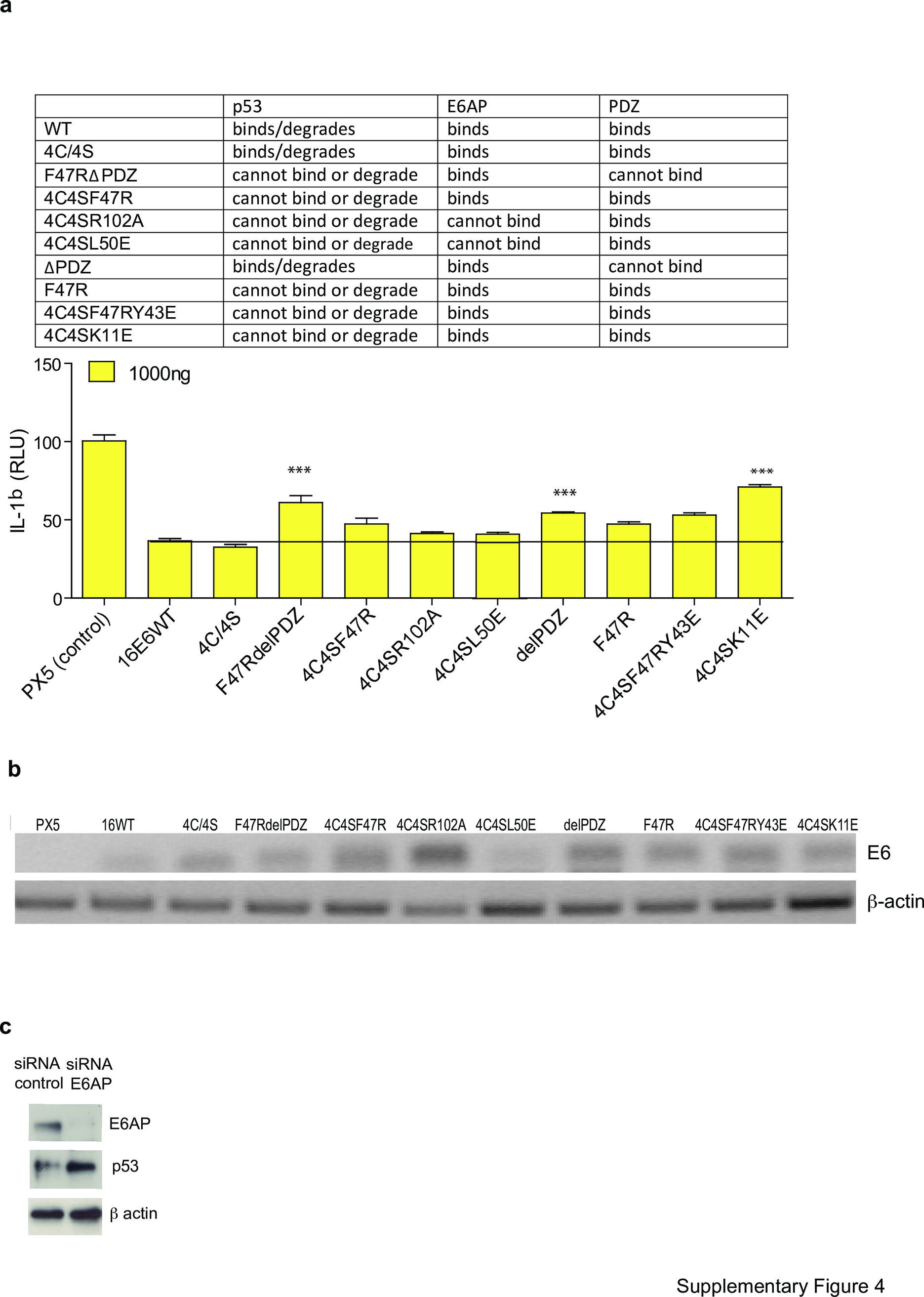

Supplement: S4 Fig — (A) Table describing 16E6 mutations. NIKs were transfected with 16E6Wt and mutations were co-transfected with IL-1β promoter luciferase construct. Forty-eight hours post transfection cells were lysed and luciferase activity measured. n = 4. (B) NIKs were transfected with WT and mutations for 16E6. Forty-eight hours post transfection proteins were probed using 16E6 antibody. n = 3. (C) Western blot to control E6AP knock down by control and SiRNA E6AP, using β-actin as a loading control. n = 4. Data are representative of n independent experiments; graphs shown are the mean ± SEM from triplicate values. (TIF) [file ppat.1007158.s004.tif]

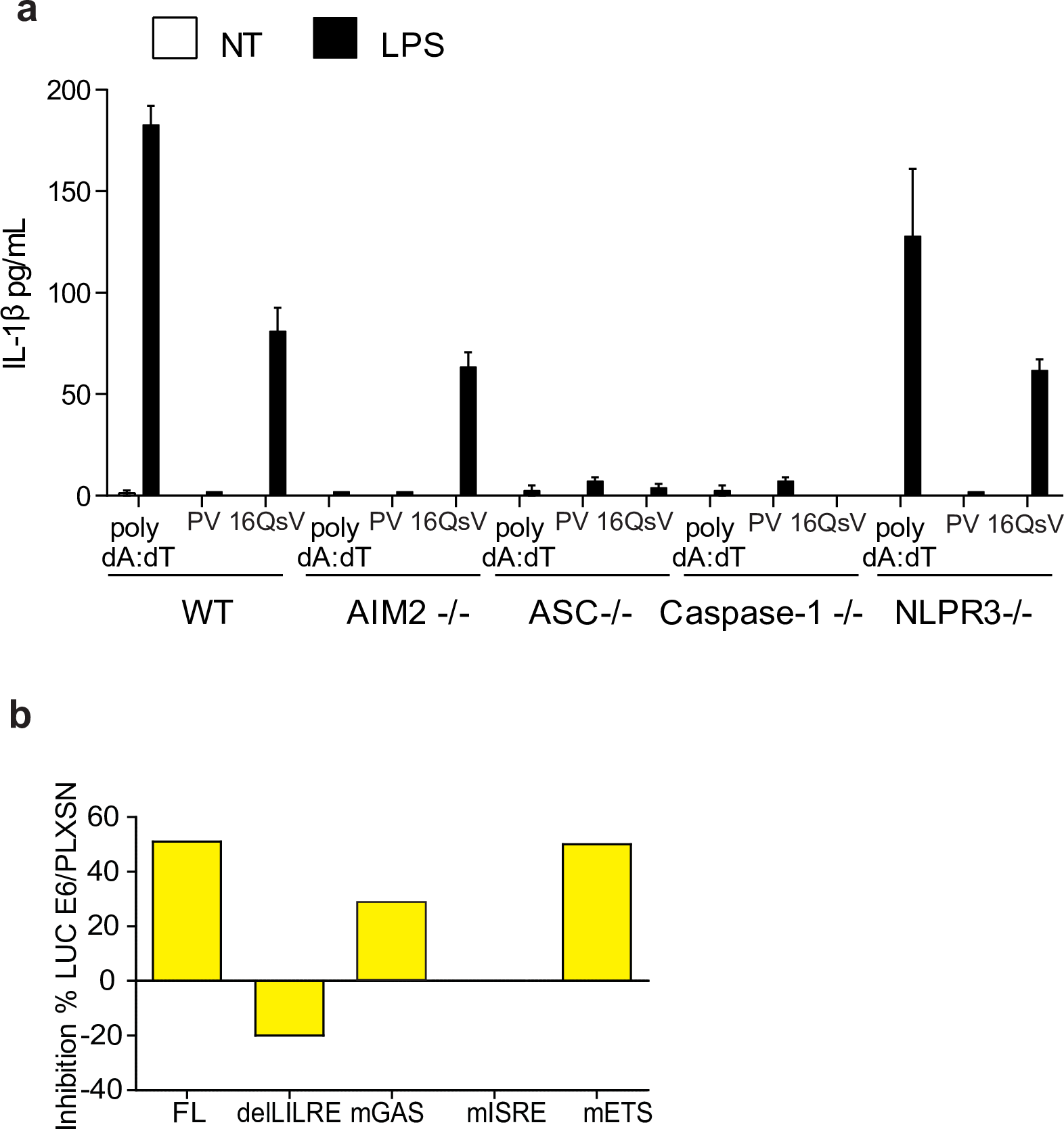

Supplement: S5 Fig — (A) 16QsV activates IL-1β production independently of AIM2 and NLRP3. Bone marrow derived macrophages from C56BL/6 WT, AIM2-/-, ASC -/-, Caspase 1 -/- (from Thomas Henry, France) and NLRP3 mice (From Virginie Petrilli, France) were isolated and cultivated as previously described [49]. (B) Percentage of IL-1β promoter inhibition with PLXSN cells vs 16E6 transfected with the IL-1β point mutation or LILRE deletion. (TIF) [file ppat.1007158.s005.tif]
